# The long isoform of FABP6 facilitates WWP2‐mediated PTEN ubiquitination to activate AKT/mTOR signalling and contributes to reduced everolimus sensitivity in clear cell renal cell carcinoma

**DOI:** 10.1002/ctm2.70880

**Published:** 2026-10-24

**Authors:** Haoming Yin, Zijie Wang, Liyuan Ge, Wei Liu, Fan Shu, Ye Yan, Hui Liang, Shudong Zhang

**Affiliations:** ^1^ Department of Urology Peking University Third Hospital Beijing China; ^2^ Department of Urology The Second Affiliated Hospital of Kunming Medical University Kunming China; ^3^ Department of Pathology School of Basic Medical Sciences Peking University Third Hospital Peking University Health Science Center Beijing China

**Keywords:** Everolimus, FABP6, PI3K‐AKT‐mTOR pathway, PTEN degradation

## Abstract

How PTEN function is impaired in clear cell renal cell carcinoma (ccRCC) without detectable genomic alterations remains unresolved. We investigated whether the long isoform of fatty acid‐binding protein 6 (FABP6L) contributes to this process. Analyses that distinguished FABP6 isoforms identified FABP6L as the principal tumour‐upregulated variant, whereas the short isoform, FABP6S, did not show a comparable increase. SHOX2 depletion preferentially reduced FABP6L expression. Biochemical experiments linked FABP6L to increased association between PTEN and the E3 ligase WWP2, enhanced PTEN polyubiquitination, and reduced PTEN protein stability. These changes were accompanied by increased AKT/mTOR signalling. Gain‐ and loss‐of‐function experiments supported a growth‐promoting role for FABP6L in cultured ccRCC cells and xenografts, while genetic rescue experiments indicated a major contribution of PTEN to the signalling and growth phenotypes. In preclinical models with reduced everolimus sensitivity, FABP6L depletion increased PTEN abundance and enhanced the response to mTOR inhibition. The findings support an isoform‐specific mechanism connecting FABP6L to PTEN turnover and ccRCC growth. They also provide a basis for investigating FABP6L‐directed approaches to improve everolimus response, although their clinical relevance requires further validation.

## Key points

- SHOX2 contributes to FABP6L upregulation in ccRCC.
- FABP6L facilitates PTEN–WWP2 association and promotes PTEN ubiquitination.
- FABP6L‐driven PTEN loss enhances AKT/mTORC1 signalling and tumour growth.
- FABP6L depletion restores PTEN and increases everolimus sensitivity in preclinical models.

## Graphical abstract

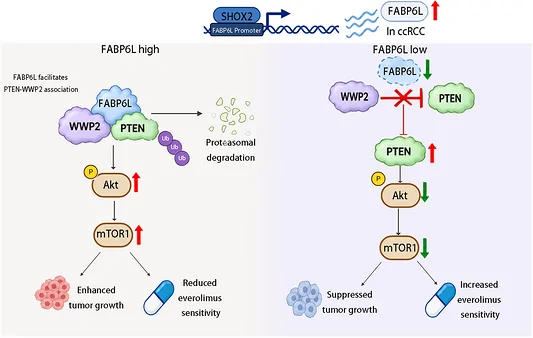

## 1 INTRODUCTION

Clear cell renal cell carcinoma (ccRCC) represents the dominant histological form of kidney cancer and frequently exhibits elevated PI3K–AKT–mTOR activity, a signalling state linked to tumour growth, metabolic adaptation, and therapeutic resistance.^1^, ^2^, ^3^, ^4^, ^5^, ^6^ PTEN is a major negative regulator of this pathway, but genetic alteration of PTEN accounts for only part of the reduction in PTEN activity observed in ccRCC.^7^, ^8^ Changes occurring after transcription are therefore likely to be important. In particular, ubiquitin‐dependent protein turnover can markedly influence PTEN abundance,^6^, ^9^, ^10^, ^11^ yet the molecular factors that determine PTEN ubiquitination in ccRCC remain incompletely understood.

Alternative isoforms generated from a single gene can acquire distinct biological functions.^12^, ^13^ FABP6 produces two principal protein forms: FABP6S and the longer FABP6L, which contains an additional N‐terminal segment. Although increased total FABP6 expression has been reported in several malignancies,^14^, ^15^, ^16^, ^17^, ^18^ most previous analyses did not resolve these isoforms. Consequently, it remains unclear which FABP6 species accounts for the expression changes and functional associations reported in tumours, including ccRCC.

In this study, we demonstrate that FABP6L, rather than the canonical FABP6S, is selectively upregulated in ccRCC and functions as an isoform‐specific regulator of PTEN‐linked AKT/mTOR signalling. Mechanistically, the unique N‐terminal extension of FABP6L supports its interaction with WWP2, while a central region contributes to PTEN binding. Together, our biochemical, genetic, and isoform‐specific analyses support a model in which FABP6L facilitates WWP2‐mediated PTEN ubiquitination and proteasomal degradation, resulting in enhanced AKT/mTOR pathway activity, tumour growth, and reduced everolimus sensitivity in preclinical models.^19^, ^20^ We therefore present FABP6L as a candidate adaptor linking non‐genetic PTEN inactivation to ccRCC progression, while acknowledging that clinical validation in treatment‐defined patient cohorts remains necessary.

## 2 MATERIALS AND METHODS

### 2.1 Cell culture and authentication

Cells used in this work included 769‐P, SN12C, A‐498, Caki‐1, 786‐O, OS‐RC‐2, HK‐2, and HEK293T, all obtained from Procell. Each line was propagated in the supplier‐recommended RPMI‐1640 or DMEM/F12 medium containing 10% FBS and 1% penicillin–streptomycin. Cultures were maintained at 37°C under 5% CO_2_. Cell identity was verified by STR analysis, and PCR screening confirmed the absence of mycoplasma (Supplementary File 1).

### 2.2 Human tissue samples

Paired tumour and non‐tumour renal tissues were obtained from 81 patients undergoing partial or radical nephrectomy at Peking University Third Hospital. Non‐tumour specimens were taken at least 2 cm from the tumour edge. Histopathology confirmed ccRCC in every case, and all samples were obtained before chemotherapy, radiotherapy, or systemic anticancer treatment. Material intended for RNA or protein extraction was immediately frozen and stored at −80°C; specimens used for histology were fixed in 4% paraformaldehyde before paraffin embedding. Ethical approval was granted under M2017147 and IRB0000‐M20260071, with written informed consent obtained from all participants.

### 2.3 Antibodies and reagents

Primary antibodies used for Western blot (WB), immunohistochemistry (IHC), immunofluorescence (IF), and immunoprecipitation (IP) are detailed in Table S2.

A custom‐made rabbit polyclonal antibody specifically targeting the unique N‐terminal extension of FABP6L (home‐made FABP6L) was generated by Biodragon using the FABP6L‐specific peptide CTWVSRKGDLQRMKQTHKGKPPSS as the immunogen. The antibody was validated using isoform‐specific overexpression lysates, peptide competition assays, and FABP6L knockdown/knockout samples. The home‐made FABP6L antibody was used at dilutions of 1:1000 for Western blotting, 1:100 for immunohistochemistry, and 1:100 for immunofluorescence. For immunoprecipitation, 2 µg of antibody was used per reaction. These applications and working dilutions or amounts are summarised in Table S2. Commercial antibodies recognising shared FABP6 regions were used only for total FABP6 detection and were not used to infer FABP6L‐specific expression.

Chemical modulators and inhibitors, including everolimus (RAD001), MK‐2206 (HY‐108232), SC‐79 (HY‐18749), MG132 (HY‐13259), chloroquine (HY‐17589A), and cycloheximide (HY‐12320), were obtained from MedChemExpress LLC.

### 2.4 Plasmids and transfection

Full‐length cDNAs encoding human FABP6L, FABP6S, PTEN, SHOX2, and WWP2 were amplified by PCR and sub‐cloned into pCDH‐CMV vectors, fused with HA, 3×Flag, Myc, His, or EGFP tags at either the N‐ or C‐terminus as indicated. FABP6L deletion mutants used for domain‐mapping experiments were generated from the full‐length FABP6L construct by PCR‐based cloning.

Sanger sequencing confirmed the identity of all constructs. Transient transfections were carried out at 70–80% confluence in six‐well plates or 10‐cm dishes, using Lipofectamine 3000 (L3000008, Invitrogen) or polyethylenimine (C0541‐1 mL, Beyotime) according to the supplier's instructions. Stable expression was established by lentiviral transduction of ccRCC cells and approximately 7 days of puromycin selection (HY‐K1057, MCE). RT‐qPCR and Western blotting were used to verify expression in the resulting cell pools or individual clones.

### 2.5 CRISPR‐Cas9 gene editing

An sgRNA directed against FABP6L (5′‐GGAGATGCAGGCGCTGACTC‐3′), selected using E‐CRISP, was cloned into lentiCRISPR v2 (Addgene #52961). Lentiviral particles were generated in HEK293T cells by cotransfection with psPAX2 and pMD2.G and were subsequently used to infect 769‐P and Caki‐1 cells in the presence of polybrene (HY‐112735). Following 7 days of puromycin selection (HY‐K1057), surviving cells were subjected to limiting dilution at approximately 0.5 cell per well in 96‐well plates. Cells carrying a non‐targeting sgRNA were maintained in parallel as controls. Putative knockout clones were first screened for loss of FABP6L protein and then genotyped by PCR amplification and Sanger sequencing across the edited locus. The selected 769‐P and Caki‐1 clones each contained a one‐nucleotide deletion that caused a frameshift and was predicted to introduce premature translation termination (Extended Data Figure 1J).

### 2.6 Total RNA isolation and quantitative Real‐Time PCR (qRT‐PCR)

Total RNA was recovered from cells or tissue specimens using TRIzol (R411‐01, Vazyme). One microgram of each preparation was used for cDNA synthesis with the PrimeScript RT Reagent Kit containing a genomic‐DNA removal step (G592, ABM). Quantitative PCR was performed on a QuantStudio 5 platform with SYBR Green Premix Ex Taq (Q712‐02, Vazyme), using the primers provided in Table S3. β‐actin served as the reference gene. Fold changes between experimental groups were derived using the 2^−ΔΔCt^ method. For comparisons of FABP6L and FABP6S abundance within the same sample, values were instead expressed as 2^−ΔCt^ using isoform‐specific primer pairs.

### 2.7 RNA sequencing and bioinformatic analysis

RNA sequencing compared FABP6L‐depleted 769‐P cells with siRNA control cells, with three independently prepared samples in each group. Library preparation and sequencing were performed by Novogene (Beijing, China) after RNA quality assessment with an Agilent 2100 Bioanalyzer. Sequencing generated paired‐end reads of 150 bases. Reads were cleaned with fastp v0.19.7 and mapped to hg38 using HISAT2 v2.0.5; featureCounts v1.5.0‐p3 was then used to obtain gene‐level counts. FPKM values were used for visualisation, whereas statistical testing of differential expression was based on raw counts in DESeq2 v1.20.0. Genes satisfying |log2 fold change| > 1 and adjusted *p* < 0.05 were considered differentially expressed. KEGG enrichment was carried out in clusterProfiler v3.8.1, with Benjamini–Hochberg‐adjusted *p* < 0.05 as the significance threshold.

### 2.8 Western blot

RIPA buffer containing protease inhibitors was used to extract proteins from cultured cells and pulverised tissues; phosphatase inhibitors were added when phosphoproteins were analysed. Samples remained on ice for 30 min, and insoluble material was removed by centrifugation (12 000 × g, 15 min, 4°C). Protein content was quantified using the BCA method. Depending on the experiment, 10–80 µg protein was separated electrophoretically and electrotransferred to 0.22‐µm PVDF. After blocking in TBST containing 5% milk or BSA, membranes were exposed to primary antibodies (Table S2) overnight at 4°C and then to HRP‐linked secondary antibodies at 1:5000 for 1 h. ECL was used for signal visualisation.

### 2.9 Cycloheximide chase assay

Protein turnover was evaluated after addition of CHX at 50 µg/mL. Lysates harvested at the indicated intervals were analysed for PTEN and β‐actin by immunoblotting. PTEN intensity was normalised to β‐actin and then to its value before CHX exposure. Decay curves and PTEN half‐life estimates were obtained by nonlinear regression in GraphPad Prism 9.0. The analysis was reproduced in at least three independent experiments.

### 2.10 RNA interference

FABP6L‐targeting RNA interference sequences were selected from its specific N‐terminal coding or transcript regions to limit effects on FABP6S (Extended Data Figure 1R). For stable depletion, the shRNA sequences and a non‐targeting control were inserted into pLKO.1‐puro. Lentivirus was produced in HEK293T cells with psPAX2 and pMD2.G, and transduced cells underwent puromycin selection (HY‐K1057, MCE) for at least 3 days.

For transient depletion, RuiBiotech supplied the siRNAs, which were delivered at 50 nM using Lipofectamine RNAiMAX (13778100, Thermo Fisher Scientific). Functional experiments were conducted after depletion had been checked by RT‐qPCR and Western blotting. Tables S4 and S5 provide the RNA interference sequences.

### 2.11 Immunohistochemistry (IHC)

Formalin‐fixed paraffin material was sectioned at 4 µm. After removal of paraffin and rehydration, antigen retrieval was carried out for 20 min in citrate buffer at pH 6.0. Sections were then treated sequentially with 3% H_2_O_2_ and 5% normal goat serum to suppress endogenous peroxidase activity and nonspecific staining, respectively. Primary antibodies (Table S2) were left on the sections overnight at 4°C, followed by a 30‐min incubation with the appropriate HRP‐linked secondary reagent. DAB was used for chromogenic detection and hematoxylin for nuclear counterstaining. For clinical specimens, FABP6L was evaluated independently by two blinded observers using H‐scores. Xenograft staining for FABP6L, PTEN, p‐AKT (Thr308), and p‐4EBP1 (Thr70) was quantified in the same manner, whereas Ki‐67 was expressed as the fraction of positively stained tumour nuclei. Several non‐overlapping microscopic fields were averaged for each tumour so that each mouse contributed a single biological value.

### 2.12 Immunoprecipitation

Endogenous IP samples were prepared using NP‐40 buffer supplemented with protease inhibitors; the buffer contained 150 mM NaCl, 5 mM EDTA, and 50 mM Tris‐HCl adjusted to pH 7.4. Tagged‐protein experiments were harvested 48 h after transfection. An aliquot corresponding to 10% of each extract was reserved as input. The remainder was incubated overnight at 4°C with 2 µg of the appropriate antibody or matched IgG. Immune material was recovered during a 4‐h incubation with Protein A/G or tag‐specific magnetic beads, washed, released into SDS sample buffer, and analysed by immunoblotting.

For sequential immunoprecipitation (Re‐IP) assays, protein complexes recovered from the first immunoprecipitation were eluted under conditions compatible with subsequent immunoprecipitation and subjected to a second immunoprecipitation using the indicated antibody or tag‐specific magnetic beads. The resulting complexes were analysed by Western blotting for the indicated proteins. Because tagged proteins were used in these experiments, these assays are referred to as exogenous or semi‐exogenous Re‐IP assays rather than fully endogenous native‐complex analyses.

### 2.13 Cell‐based ubiquitination assay

Cells expressing Flag‐PTEN and ubiquitin were subjected to the indicated FABP6L, FABP6S, WWP2, siRNA, or deletion‐mutant conditions. MG132 exposure (10 µM for 8 h; HY‐13259, MCE) preceded collection. Denaturing lysis was used to dissociate noncovalently associated proteins, after which the lysates were diluted to allow recovery of Flag‐PTEN on anti‐Flag magnetic beads. Anti‐ubiquitin immunoblotting detected ubiquitin conjugates associated with the recovered PTEN. Input samples were analysed for PTEN, the relevant FABP6 isoform, WWP2, and β‐actin.

### 2.14 Mass spectrometry

Caki‐1 cells stably expressing FABP6L‐3×Flag or empty vector provided the samples for Co‐IP/MS. Immunoprecipitates were resolved on 10% SDS‐PAGE gels and silver‐stained (24612, Thermo Fisher Scientific). Each lane was divided into slices for in‐gel processing. Reduction with 10 mM DTT and alkylation with 55 mM IAA preceded trypsin digestion. Extracted peptides were desalted on ZipTip C18 columns and analysed with an EASY‐nLC 1200 coupled to a Q Exactive HF mass spectrometer (Thermo Fisher Scientific).

MaxQuant v1.6.0 was used to process the spectra against the UniProt human proteome database. Candidate associated proteins were selected from the label‐free comparison of FABP6L‐3×Flag and empty‐vector samples. Selection required at least two unique peptides and an LFQ intensity ratio exceeding 2.0 for FABP6L‐3×Flag relative to the control.

The mass spectrometry proteomics data have been deposited in the ProteomeXchange Consortium via the iProX partner repository with the dataset identifier PXD077055 and iProX project identifier IPX0016559000. The dataset is publicly available through ProteomeXchange at http://proteomecentral.org/cgi/GetDataset?ID=PXD077055  and through iProX at https://www.iprox.cn/page/project.html?id=IPX0016559000 .

### 2.15 Immunofluorescence

Cells were grown on coverslips in 24‐well plates to 50%–70% confluence. Where exogenous proteins were examined, the specified FABP6L‐HA, PTEN‐EGFP, and WWP2‐Flag constructs were introduced 48 h before fixation. Following fixation with 4% paraformaldehyde for 15 min, coverslips were permeabilised for 10 min in 0.1% Triton X‐100, rinsed with PBS, and blocked in 5% BSA for 1 h.

The specimens were exposed to the indicated primary antibodies at 4°C overnight and subsequently to Alexa Fluor 488‐ or 594‐conjugated secondary antibodies for 1 h at room temperature. DAPI staining (D9542‐1MG, Sigma‐Aldrich) lasted 5 min. Coverslips were mounted in an anti‐fade medium, and representative fields were imaged with an Olympus confocal laser scanning microscope.

Colocalisation was analysed using ImageJ. Pearson's correlation coefficients (PCCs) were calculated from at least ten randomly selected fields per group. For fluorescence intensity profile analysis, a line was drawn across representative cells using the same image‐analysis procedure for each group, and the fluorescence intensity of each channel along the selected line was extracted using the Plot Profile function in ImageJ. The resulting intensity profiles were plotted as a function of distance along the selected line.

### 2.16 Oil Red O staining

Neutral lipid staining was performed with the Modified Oil Red O kit (60720ES50, Yeasen). Kit components A1 and A2 were combined at 3:2, allowed to stand for 10 min, and filtered before use. PBS‐washed cells underwent fixation with 4% paraformaldehyde for 20 min, brief treatment with 60% isopropanol, and Oil Red O staining for 15 min. Residual dye was removed with 60% isopropanol before hematoxylin counterstaining. The stained area was quantified from light‐microscopy images using ImageJ and normalised to the corresponding cell‐associated area.

### 2.17 Colony formation assay

For clonogenic growth, 500 cells were placed in each well of a six‐well plate, with three wells used for every condition. Cultures were continued for 14 days with medium replacement every 3 days. Once colonies were readily visible, cells were washed, fixed in methanol for 20 min, and exposed to 0.5% crystal violet for 30 min. After removal of excess stain and air drying, plates were photographed and colony numbers were obtained using ImageJ.

### 2.18 Cell viability and everolimus dose–response assays

For viability measurements, 1000 cells were seeded in 100 µL complete medium per well of a 96‐well plate. Growth was followed at the indicated time points. For drug‐response experiments, cells were exposed to graded concentrations of everolimus for 72 h. CCK‐8 reagent (10 µL) was then added to each well and allowed to react for 2 h at 37°C before absorbance was read at 450 nm. Concentration‐response data were fitted in GraphPad Prism 9.0 to calculate IC50 estimates and their 95% confidence intervals. Each condition included at least three replicate wells, and experiments were repeated independently at least three times.

### 2.19 EdU cell proliferation assay

Cells were labelled with 10 µM EdU for 4 h and subsequently processed for fluorescence detection. Fixation was performed with 4% paraformaldehyde for 15 min, followed by a 20‐min permeabilisation step in 0.5% Triton X‐100. The supplied click‐reaction mixture was applied for 30 min in darkness, and nuclei were subsequently labelled with DAPI. Five randomly selected fields were recorded from each well. The EdU‐labelling index was calculated as 100 × EdU‐positive nuclei/DAPI‐positive nuclei. Quantification was based on three independent experiments.

### 2.20 Chromatin immunoprecipitation (ChIP) assay

SHOX2 binding to the FABP6L promoter was evaluated using the Beyotime ChIP kit (P2078). Cells were first fixed for 10 min with 1% formaldehyde, after which glycine was added to 0.125 M to terminate cross‐linking. Chromatin released in the kit lysis buffer was sheared by sonication to a predominant size range of approximately 200–500 bp. Clarified chromatin was divided between anti‐SHOX2 antibody (5 µg; sc‐81955) and species‐matched IgG and incubated at 4°C overnight. Protein A/G magnetic beads were used to isolate antibody‐associated chromatin. Following the kit washing and elution steps, cross‐links were removed at 65°C for 4 h and DNA was purified. qPCR was then used to quantify the FABP6L promoter region and the negative‐control locus; primer sequences are provided in Table S3.

### 2.21 Dual luciferase reporter assay

A PCR‐amplified segment of the human FABP6L promoter encompassing the predicted binding sites was inserted into pGL3‐Basic (212936, Addgene). Reporter experiments used 293T cells plated at 2 × 10^4^ cells per well in 24‐well plates. Each transfection combined 200 ng promoter reporter, 20 ng pRL‐TK (Promega), and 50 nM siRNA directed against MIXL1, NKX2‐5, WT1, or SHOX2; a non‐targeting siRNA provided the control. Lipofectamine 3000 (L3000001, Invitrogen) was used for delivery.

Luciferase activities were recorded 48 h later, and promoter activity was expressed as the ratio of firefly to Renilla signal (DL101‐01, Vazyme). Three technical measurements were included per experiment, and the experiment was independently repeated at least three times.

### 2.22 Xenograft mouse models

Animal experiments were approved by the Peking University Health Science Center IACUC (DLASBD0141) and were conducted in accordance with applicable institutional and NIH animal‐welfare requirements. Female BALB/c nude mice, 4–6 weeks of age and weighing 18–22 g, were housed under SPF conditions with a 12‐h light/dark cycle. Tumours were initiated by subcutaneous injection of 5 × 10^6^ cells in 100 µL of a 1:1 PBS/Matrigel mixture into the right flank. Calliper measurements were collected every 3 days, and tumour volume was calculated as length × width^2^/2.

Everolimus was freshly diluted from a DMSO stock into 0.5% CMC‐Na and administered by oral gavage once daily at 3 mg/kg; control mice received the corresponding vehicle.

For the everolimus‐resistant Caki‐1 xenograft model, everolimus‐resistant Caki‐1 cells with wild‐type FABP6L or FABP6L knockout were implanted subcutaneously into nude mice. Mice were assigned to four groups: FABP6L‐WT plus vehicle, FABP6L‐WT plus everolimus, FABP6L‐KO plus vehicle, and FABP6L‐KO plus everolimus. Everolimus treatment was initiated on day 21 after tumour‐cell inoculation. Tumour dimensions were measured every 3 days using digital callipers. Mice were euthanised on day 41 after tumour‐cell inoculation, and tumours were excised, photographed, and weighed.

For the parental Caki‐1 xenograft model, parental Caki‐1 cells stably expressing empty vector or FABP6L were implanted subcutaneously using the same procedure. When tumours reached an average volume of approximately 100 mm^3^ on day 13 after tumour‐cell inoculation, mice were randomly assigned to four groups with five mice per group: EV plus vehicle, EV plus everolimus, FABP6L‐OE plus vehicle, and FABP6L‐OE plus everolimus. Everolimus was administered by oral gavage at 3 mg/kg once daily. Vehicle‐treated mice received an equivalent volume of the corresponding vehicle. Tumour dimensions and body weights were monitored throughout the treatment period. Mice were euthanised on day 33 after tumour‐cell inoculation, and tumours were excised, photographed, and weighed.

### 2.23 Generation of everolimus‐resistant Caki‐1 cells

Everolimus‐resistant Caki‐1 cells were generated by long‐term stepwise exposure to increasing concentrations of everolimus. Parental Caki‐1 cells were initially exposed to 0.5 µM everolimus. After the cells adapted and resumed stable proliferation, the drug concentration was progressively increased to 1, 2, 4, and 8 µM. Cells that maintained stable growth in 8 µM everolimus were designated everolimus‐resistant Caki‐1 cells (everolimus‐R) and were subsequently maintained at this concentration. Before drug‐response assays, resistant cells were cultured in drug‐free medium for 2 days. Resistance was confirmed by CCK‐8‐based dose–response analysis, showing reduced sensitivity to everolimus compared with parental Caki‐1 cells.

### 2.24 Public data resources

The accession numbers, sample information, and analysis purposes of the public datasets used in this study are provided in the corresponding figure legends and supplementary tables. GEO datasets GSE53757, GSE40435, GSE16441, GSE16449, and GSE36895 were used for paired tumour–normal discovery analyses. TCGA‐KIRC, GEPIA, UALCAN, TISCH, and AnimalTFDB were used for validation, survival, proteomic, single‐cell, and transcription‐factor screening analyses, as indicated. For resources that did not distinguish FABP6 isoforms, the corresponding analyses were interpreted as total FABP6 rather than FABP6L‐specific signals.

### 2.25 Statistical analysis

Statistical procedures were selected according to the experimental design and, where sample size permitted, the underlying data distribution. Two independent groups were compared using a two‐tailed Student's *t*‐test or a nonparametric counterpart. One‐way ANOVA followed by Tukey's testing was used for appropriate multi‐group comparisons, whereas two‐way ANOVA was applied to factorial or treatment‐by‐time designs. Serial xenograft‐volume measurements were analysed by repeated‐measures two‐way ANOVA. Kaplan–Meier curves were compared using the log‐rank test, and associations between continuous variables were assessed with Spearman correlation unless otherwise specified. IC50 values and 95% confidence intervals were obtained by nonlinear regression. An extra sum‐of‐squares *F* test was used where protein‐decay models were compared. All statistical tests were two‐sided where applicable, and significance was assigned when *p* was below 0.05.

## 3 RESULTS

### 3.1 Result 1: Isoform‐resolved analysis identifies FABP6L as the predominant FABP6 variant overexpressed in ccRCC

To identify candidate oncogenic drivers associated with clear cell renal cell carcinoma (ccRCC) progression, we performed differential expression analysis across five independent GEO datasets (GSE53757, GSE40435, GSE16441, GSE16449, and GSE36895), each containing paired tumour and adjacent normal tissues^21^ (Figure 1A and B). These paired cohorts were used as the discovery datasets to reduce inter‐individual variability in tumour‐normal comparisons. Using the criteria of |logFC| > 1 and adjusted *p* <  0.01, we identified 190 genes that were consistently upregulated across the five datasets. Subsequent survival analysis of these candidates using the log‐rank test identified FABP6 as the top‐ranked candidate associated with unfavourable overall survival (Table S1). According to NCBI RefSeq database, the FABP6 gene locus encodes two distinct protein isoforms: a short isoform FABP6S (128 aa), and a long isoform FABP6L (177 aa) characterised by a unique 49‐amino‐acid N‐terminal extension^12^ (Figure 1C and D).

**FIGURE 1:**
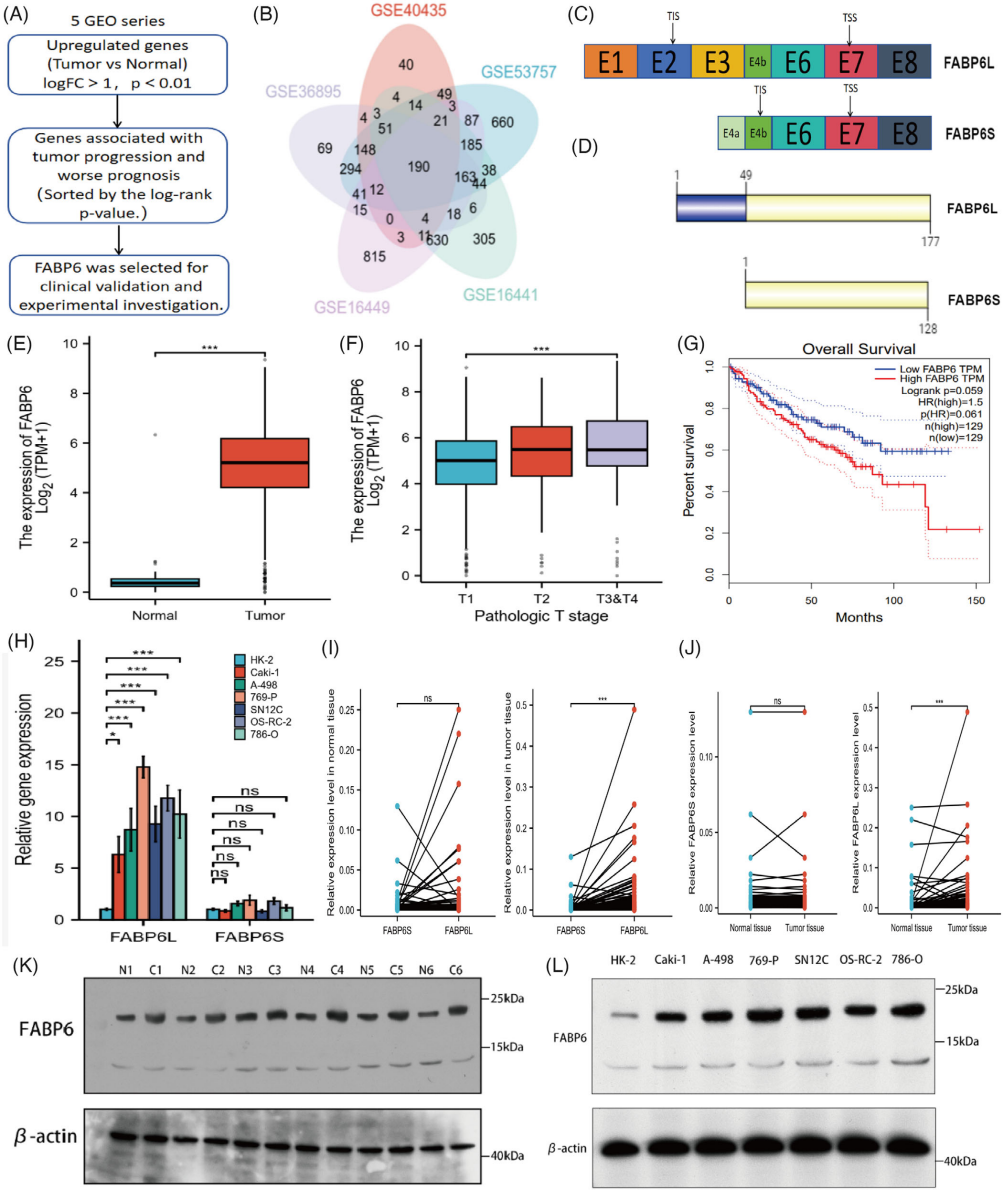
Isoform‐resolved analysis identifies FABP6L as the predominant FABP6 variant overexpressed in ccRCC. (A) Schematic workflow for the identification of candidate genes. Five GEO datasets were analysed to identify upregulated genes (|logFC| > 1, adjusted *p* < 0.01) associated with tumour progression and poor prognosis, leading to the selection of FABP6. (B) Venn diagram showing the overlap of significantly upregulated genes across five independent GEO cohorts (GSE36895, GSE40435, GSE53757, GSE16441, and GSE16449). 190 genes were consistently upregulated. (C, D) Schematic representation of the mRNA structure (C) and protein domains (D) of the two FABP6 isoforms: the long isoform (FABP6L, 177 aa) and the short isoform (FABP6S, 128 aa). FABP6L contains a unique 49‐amino‐acid N‐terminal extension. (E) Comparison of total *FABP6* mRNA expression between normal and tumour tissues based on TCGA/GTEx data. (F) Correlation between total FABP6 expression and pathologic T stage (T1, T2, and T3&T4) in ccRCC patients. (G) Kaplan–Meier survival analysis according to total FABP6 expression (red) and overall survival (OS) in ccRCC patients (*p* = 0.059 by log‐rank test; HR = 1.5). (H) RT‐qPCR analysis of *FABP6L* and *FABP6S* mRNA levels in a panel of ccRCC cell lines (Caki‐1, A‐498, 769‐P, SN12C, OS‐RC‐2, 786‐O) compared to normal renal tubular cells (HK‐2). (I) Isoform‐specific RT‐qPCR analysis of *FABP6S* and *FABP6L* mRNA expression in paired adjacent normal and ccRCC tissues. In adjacent normal tissues, FABP6S and FABP6L showed comparable expression, whereas FABP6L expression was significantly higher than FABP6S in ccRCC tissues. (J) Paired comparison of *FABP6S* and *FABP6L* mRNA expression between ccRCC tissues and matched adjacent normal tissues. FABP6S expression showed no significant difference between tumour and adjacent normal tissues, whereas FABP6L was significantly upregulated in ccRCC tissues. (K) Western blot analysis of FABP6 protein levels in six pairs of clinical ccRCC tissues (C) and their matched adjacent normal tissues (N). β‐actin served as the loading control. (L) Western blot analysis of FABP6 protein levels across the HK‐2 cell line and various ccRCC cell lines. The band corresponding to the higher molecular weight (FABP6L) is the predominant form in cancer cells. Data are presented as mean ± SD. *p < 0.05, **p < 0.01, ***p < 0.001.

Validation using the GEPIA database corroborated that *FABP6* mRNA is markedly elevated in ccRCC tissues compared with normal controls (Figure 1E). Notably, *FABP6* expression correlated positively with advanced T‐stage (Figure 1F), but was not significantly associated with M stage ^22^ (Extended Data Figure1A). Kaplan–Meier analysis showed a trend towards poorer overall survival in patients with higher total FABP6 expression ^23^ (Figure 1G). At the protein level, analysis via the UALCAN portal further confirmed elevated FABP6 abundance in ccRCC tissues, with expression increasing alongside tumour stage and grade ^24^ (Extended Data Figure1B–D). Furthermore, single‐cell transcriptomic analysis via the TISCH database revealed that FABP6 expression is explicitly compartmentalised within malignant epithelial cells, with minimal expression/negligible signals in stromal or immune compartments ^25^ (Extended Data Figure1E). Collectively, these findings implicate FABP6 as a candidate associated with ccRCC progression.

Since the public datasets referenced above do not reliably distinguish between the two FABP6 isoforms, we next sought to determine which isoform accounted for the tumour‐associated increase observed in ccRCC. RT‐qPCR analysis across a panel of ccRCC cell lines together with immortalised normal renal tubular cells (HK‐2) showed that FABP6L was markedly upregulated in malignant cells and was more abundant than FABP6S (Figure 1H). We next examined the two isoforms in 81 paired clinical specimens. In adjacent normal tissues, *FABP6L* and *FABP6S* showed comparable mRNA expression, with no statistically significant difference between the two isoforms. In contrast, FABP6L expression was significantly higher than FABP6S expression in ccRCC tissues (Figure 1I). Consistently, paired tumour–normal analysis showed no significant change in *FABP6S* mRNA expression between ccRCC and adjacent normal tissues, whereas FABP6L was significantly upregulated in tumour tissues (Figure 1J). At the protein level, Western blotting of paired clinical specimens showed that FABP6L was enriched in tumour tissues relative to adjacent normal counterparts (Figure 1K). Western blot analysis across the cell‐line panel further showed that FABP6L was the predominant protein isoform in ccRCC cells (Figure 1L). Together, these results indicate that FABP6L is the predominant FABP6 isoform contributing to the increased FABP6 signal observed in ccRCC.

Because commercially available FABP6 antibodies recognise epitopes shared by both isoforms, we generated a custom rabbit polyclonal antibody, designated home‐made FABP6L (home‐FABP6L), against the unique N‐terminal extension of FABP6L to enable isoform‐specific detection ^12^ (Extended Data Figure 1F and G). Immunohistochemical (IHC) analysis of human ccRCC specimens using the home‐made FABP6L‐specific antibody showed stronger FABP6L staining in tumour tissues than in adjacent normal tissues. Consistently, quantitative H‐score analysis of the 81 paired specimens confirmed significantly higher FABP6L protein abundance in ccRCC tissues (Extended Data Figure 1H). Kaplan–Meier analysis further evaluated the association between FABP6L protein expression and overall survival (Extended Data Figure 1I). Collectively, these data establish FABP6L, rather than FABP6S, as the predominant and clinically relevant isoform upregulated in ccRCC.

### 3.2 Result 2: FABP6L promotes ccRCC cell proliferation and tumour growth

To investigate the functional role of FABP6L in ccRCC proliferation, we silenced FABP6L using shRNA in SN12C and 769‐P cell lines, both of which exhibit high endogenous FABP6L expression. EdU incorporation assays (Figure 2B and E), colony formation assays (Figure 2C and F), and CCK‐8 cell viability assays (Figure 2A and D) consistently revealed that FABP6L knockdown markedly reduced DNA synthesis, impaired colony forming capacity, and suppressed cell growth rates. Phenotypic consistency was further verified in 769‐P cells bearing CRISPR‐Cas9‐mediated FABP6L knockout, with knockout clones validated by Western blotting and Sanger sequencing of the targeted genomic region (Extended Data Figure1J–N). Caki‐1 and A‐498 cells with relatively low endogenous FABP6L were transduced with empty vector, FABP6S, or FABP6L to compare the functional effects of the two isoforms. FABP6L overexpression significantly enhanced cell proliferation, as evidenced by increased CCK‐8 signals (Figure 2G and J), EdU incorporation (Figure 2H and K), and colony formation (Figure 2I and L). In contrast, FABP6S overexpression did not produce a comparable proliferative effect and showed no significant increase compared with empty‐vector controls. These findings support an isoform‐specific growth‐promoting role for FABP6L in ccRCC cells.

**FIGURE 2:**
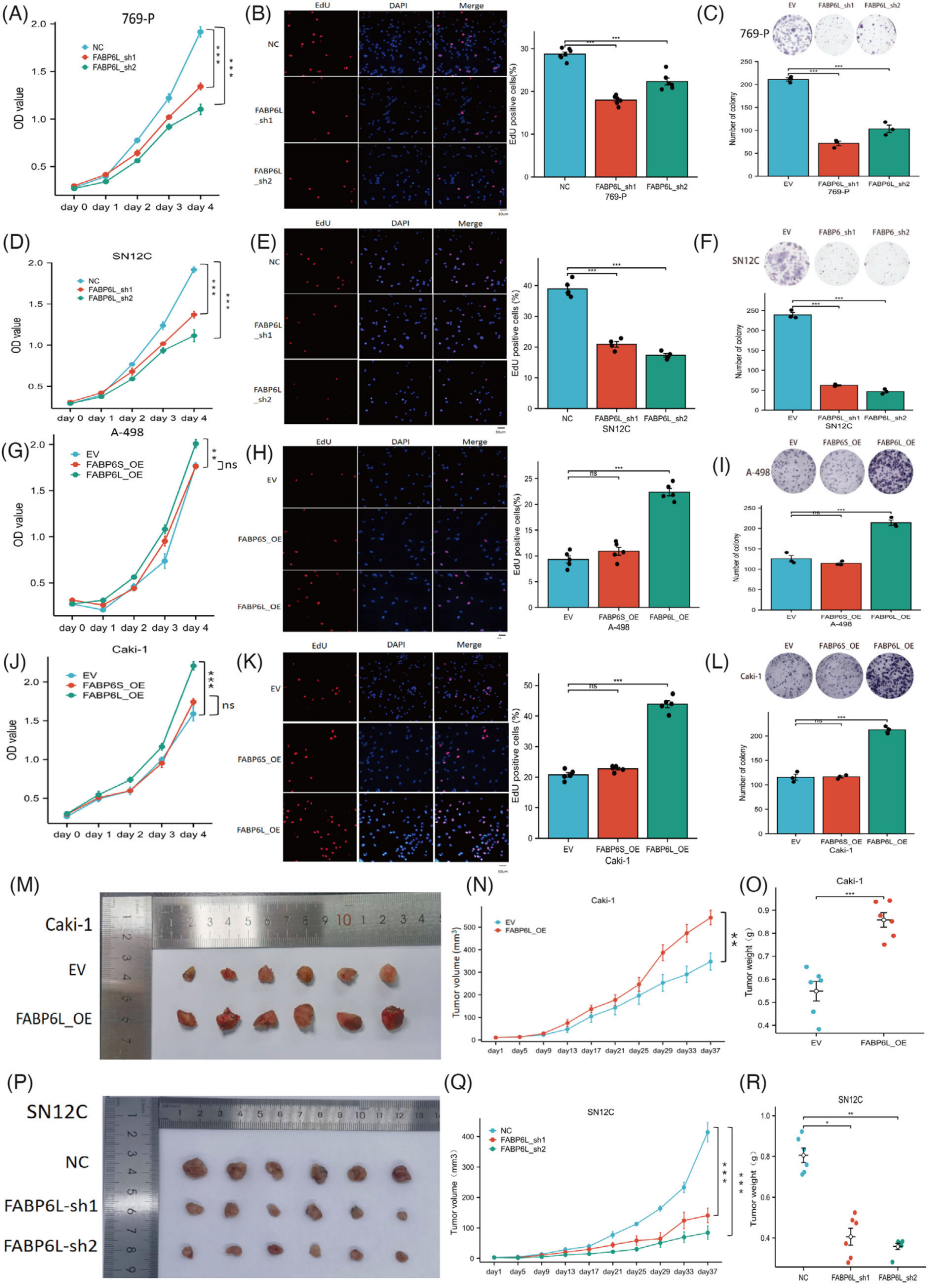
FABP6L promotes ccRCC cell proliferation and tumour growth. (A–F) FABP6L knockdown inhibits proliferation in high‐expressing ccRCC cells. (A, D) CCK‐8 assays showing the viability of 769‐P (A) and SN12C (D) cells over 4 days following FABP6L knockdown (sh‐1, sh‐2) compared to negative control (NC). (B, E) Representative images and quantification of EdU incorporation assays in 769‐P (B) and SN12C (E) cells. Red indicates EdU‐positive proliferating cells; Blue indicates DAPI‐stained nuclei. Scale bars = 50 µm. (C, F) Colony formation assays for 769‐P (C) and SN12C (F) cells. Top, representative images of crystal violet‐stained colonies; bottom, quantification of colony numbers. (G–L) Comparison of the effects of FABP6S and FABP6L overexpression on ccRCC cell proliferation. (G, J) CCK‐8 assays of A‐498 (G) and Caki‐1 (J) cells expressing empty vector (EV), FABP6S, or FABP6L. (H, K) Representative images and quantification of EdU incorporation in A‐498 (H) and Caki‐1 (K) cells expressing EV, FABP6S, or FABP6L. Scale bars = 50 µm. (I, L) Representative images and quantification of colony formation in A‐498 (I) and Caki‐1 (L) cells expressing EV, FABP6S, or FABP6L. (M–R) FABP6L modulates ccRCC tumour growth in subcutaneous xenograft models. (M) Representative images of xenograft tumours derived from Caki‐1 cells with FABP6L overexpression (OE) versus empty vector (EV) (*n* = 6 per group). (N, O) Tumour growth curves (volume, mm^3^) (N) and terminal tumour weights (g) (O) for the Caki‐1 xenograft model. (P) Representative images of xenograft tumours derived from SN12C cells with FABP6L knockdown (sh‐1, sh‐2) versus negative control (NC) (*n* = 6 per group). (Q, R) Tumour growth curves (Q) and terminal tumour weights (R) for the SN12C xenograft model showing significant growth inhibition upon FABP6L silencing. Data are presented as mean ± SD. *p < 0.05, **p < 0.01, ***p < 0.001.

To evaluate the oncogenic relevance of FABP6L in vivo, we conducted subcutaneous xenograft experiments in nude mice. Tumours derived from FABP6L‐silenced SN12C cells showed reduced volume and weight compared with control xenografts (Figure 2P–R). In contrast, Caki‐1 cells engineered to stably overexpress FABP6L generated larger and heavier tumours than empty‐vector controls (Figure 2M–O). IHC analysis of the xenograft tumours further supported these findings. FABP6L overexpression in Caki‐1 xenografts was associated with an increased Ki‐67 proliferation index and enhanced p‐AKT (Thr308) staining, accompanied by reduced PTEN staining. Conversely, FABP6L knockdown in SN12C xenografts reduced Ki‐67 and p‐AKT (Thr308) staining and increased PTEN abundance. Quantitative analysis of the corresponding IHC staining confirmed these changes (Extended Data Figure1O and P). These in vivo experiments support a growth‐promoting role for FABP6L in ccRCC.

### 3.3 Result 3: FABP6L promotes ccRCC progression by activating the PI3K/AKT signalling pathway

To identify pathway‐level alterations associated with FABP6L depletion, we performed RNA‐seq profiling of 769‐P cells transfected with a FABP6L‐targeting siRNA or the corresponding control siRNA, with three independent biological replicates per condition. KEGG pathway enrichment analysis of the resulting differentially expressed genes highlighted the PI3K–AKT signalling pathway (Figure 3A). ^26^, ^27^ Subsequent RT‐qPCR validation confirmed that the mRNA levels of key pathway components, including *PIK3CA*, *AKT1*, *PDK1*, and *mTOR*, were significantly downregulated in FABP6L‐knockdown cells (Figure 3B). Because FABP6L is not a canonical transcription factor, these transcriptomic alterations were interpreted as pathway‐level changes rather than evidence of direct transcriptional regulation. We therefore next examined PI3K‐AKT pathway activity at the protein level.

**FIGURE 3:**
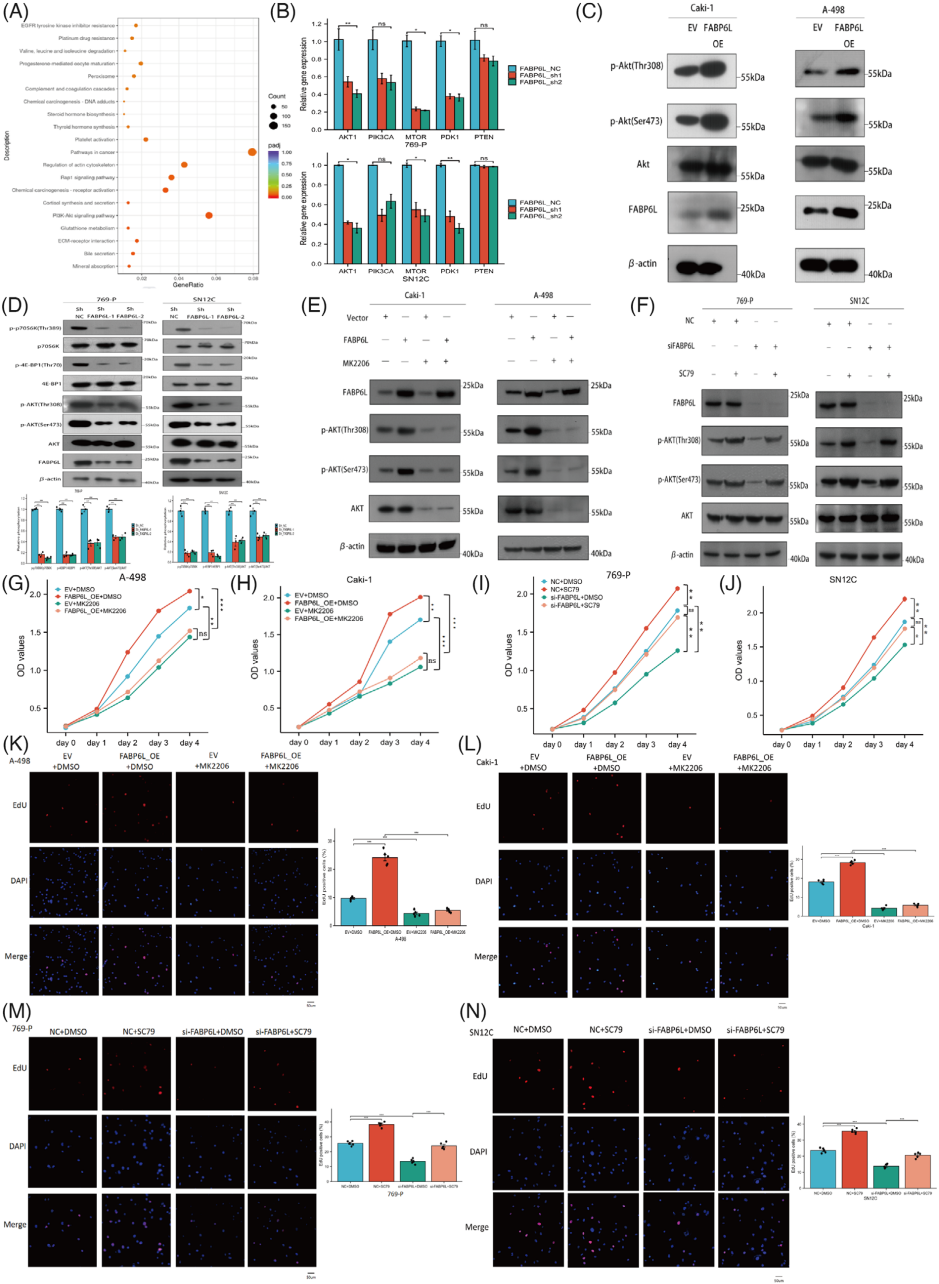
FABP6L promotes ccRCC progression by activating the PI3K/AKT signalling pathway. (A) KEGG pathway enrichment analysis of differentially expressed genes (DEGs) identified by RNA‐seq in FABP6L‐silenced 769‐P cells versus control cells (*n* = 3 independent biological replicates per condition). (B) RT‐qPCR validation of key PI3K‐AKT pathway components (AKT1, PIK3CA, MTOR, PDK1, and PTEN) in 769‐P (top) and SN12C (bottom) cells after FABP6L silencing (sh‐1, sh‐2). ns, not significant. (C) Western blot analysis of AKT phosphorylation at Thr308 and Ser473 and total AKT in Caki‐1 and A‐498 cells following FABP6L overexpression. β‐actin served as the loading control. (D) Representative Western blots of AKT/mTORC1 signalling in 769‐P and SN12C cells expressing control shRNA or two independent FABP6L‐targeting shRNAs. Phosphorylated AKT (Thr308 and Ser473), p70S6K (Thr389), and 4EBP1 (Thr70), together with their corresponding total proteins and FABP6L, were examined. β‐actin served as the loading control. (E) Western blot analysis showing that the AKT inhibitor MK‐2206 (5 µM) effectively attenuates the increase in p‐AKT (Thr308 / Ser473) induced by FABP6L overexpression in Caki‐1 and A‐498 cells. (F) Western blot analysis showing that the AKT activator SC79 (5 µg/mL) partially restores p‐AKT levels in FABP6L‐silenced (si‐FABP6L) 769‐P and SN12C cells. (G, H) CCK‐8 assays demonstrating that MK‐2206 treatment abolishes the pro‐proliferative effect of FABP6L overexpression in A‐498 (G) and Caki‐1 (H) cells. (I, J) CCK‐8 assays demonstrating that SC79 treatment rescues the impaired proliferation caused by FABP6L knockdown in 769‐P (I) and SN12C (J) cells. (K, L) Representative EdU incorporation images and quantification showing that the AKT inhibitor MK‐2206 suppresses the increased DNA synthesis induced by FABP6L overexpression in A‐498 (K) and Caki‐1 (L) cells. Scale bars = 50 µm. (M, N) Representative EdU incorporation images and quantification showing that the AKT activator SC79 restores the proliferative capacity of FABP6L‐knockdown 769‐P (M) and SN12C (N) cells. Scale bars = 50 µm. Data are presented as mean ± SD from three independent experiments. *p < 0.05, **p < 0.01, ***p < 0.001.

To assess pathway activation at the biochemical level, we analysed PI3K‐AKT signalling across four ccRCC cell lines (Caki‐1, A‐498, 769‐P, and SN12C). Western blot analysis demonstrated that ectopic expression of FABP6L in Caki‐1 and A‐498 cells significantly increased AKT phosphorylation at Ser473 and Thr308 without affecting total AKT protein levels (Figure 3C). Conversely, FABP6L knockdown in 769‐P and SN12C cells reduced AKT phosphorylation at Thr308 and Ser473, together with decreased phosphorylation of the downstream mTORC1 effectors p70S6K at Thr389 and 4EBP1 at Thr70 (Figure 3D). To further assess downstream mTORC1 signalling under basal conditions, we compared Caki‐1 and A‐498 cells expressing empty vector, FABP6S, or FABP6L. FABP6L overexpression increased phosphorylation of the mTORC1 downstream effectors p70S6K at Thr389 and 4EBP1 at Thr70, whereas FABP6S overexpression did not produce comparable changes (Extended Data Figure 1Q). These findings further support an isoform‐specific role of FABP6L in enhancing basal AKT–mTORC1 pathway activity.

To determine whether FABP6L‐driven ccRCC proliferation is functionally linked to PI3K‐AKT signalling, we performed pharmacological rescue experiments. Treatment of FABP6L‐knockdown 769‐P and SN12C cells with the AKT activator SC‐79 efficiently restored cell proliferative capacity, as measured by EdU incorporation (Figure 3M and N), and CCK‐8 assays (Figure 3I–J), accompanied by restoration of p‐AKT levels (Figure 3F). Complementarily, the pro‐proliferative effects elicited by FABP6L overexpression in Caki‐1 and A‐498 cells were effectively neutralised by the selective AKT inhibitor MK‐2206 (Figure 3E, G, H, K, and L). Together, these results indicate that FABP6L promotes ccRCC cell proliferation at least in part through activation of AKT signalling.

### 3.4 Result 4: SHOX2 contributes to FABP6L upregulation and FABP6L promotes ubiquitin‐proteasome‐mediated PTEN degradation in ccRCC

To explore candidate upstream regulators contributing to FABP6L upregulation in ccRCC, we intersected survival‐associated transcription factors (log rank *p* < 0.01) predicted by Animal TFDB 4.0 with genes more highly expressed in ccRCC than in adjacent normal tissues (logFC > 1.5) ^28^ (Figure 4A). Four candidate transcription factors emerged: MIXL1, NKX2‐5, SHOX2, and WT1, all of which showed increased expression with advancing clinical stage ^29^, ^30^ (Extended Data Figure2A–L). Dual‐luciferase reporter assays revealed that only the knockdown of SHOX2 led to a significant reduction in FABP6L promoter‐driven luciferase activity (Figure 4B), supporting SHOX2 as a candidate transcriptional regulator of FABP6L. To further examine SHOX2 occupancy at the FABP6L promoter, chromatin immunoprecipitation (ChIP) assays were performed, which demonstrated robust enrichment of SHOX2 at the FABP6L promoter region, whereas no enrichment was observed at the distal negative‐control region (Figure 4C). Isoform‐specific RT‐qPCR analysis in 769‐P and SN12C cells showed that SHOX2 depletion significantly reduced *FABP6L* mRNA expression, whereas FABP6S expression remained largely unchanged (Figure 4D and E). Consistently, Western blot analysis showed a marked reduction in FABP6L protein following SHOX2 knockdown, with no appreciable change in FABP6S protein levels (Figure 4F). Together, these findings support SHOX2 as an upstream regulator contributing preferentially to FABP6L expression in ccRCC.

**FIGURE 4:**
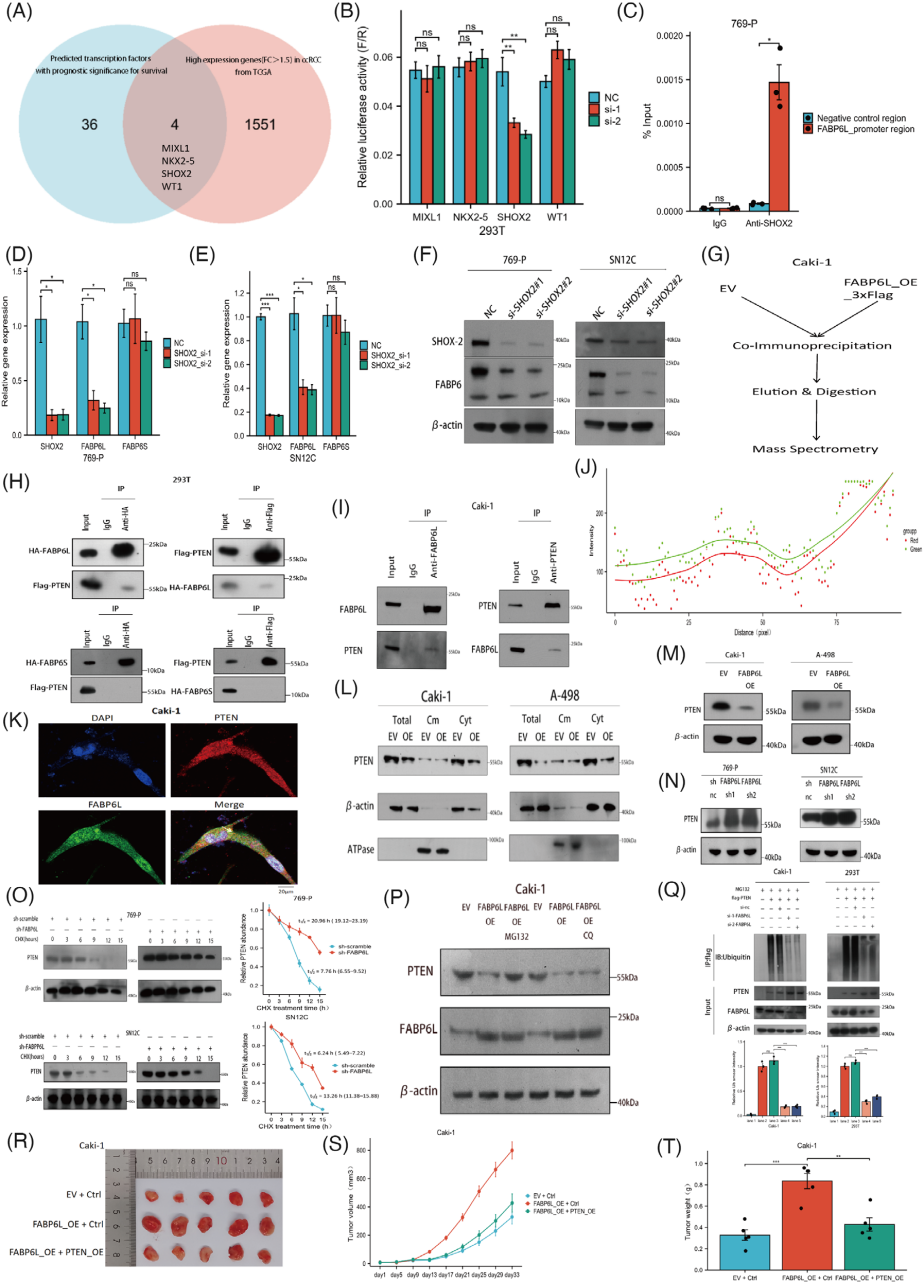
SHOX2 contributes to FABP6L upregulation and FABP6L promotes ubiquitin–proteasome‐mediated PTEN degradation in ccRCC. (A) Venn diagram showing the intersection of predicted survival‐associated transcription factors (Animal TFDB 4.0) and genes upregulated in ccRCC (TCGA). (B) Dual‐luciferase reporter assay in 293T cells showing the effect of knocking down candidate transcription factors (MIXL1, NKX2‐5, SHOX2, and WT1) on FABP6L promoter activity. **p < .01; ns, not significant. (C) ChIP‐qPCR assay showing SHOX2 enrichment at the FABP6L promoter region in 769‐P cells, with the indicated negative‐control region included to assess binding specificity. (D, E) RT‐qPCR analysis of SHOX2, FABP6L, and FABP6S expression in 769‐P (D) and SN12C (E) cells following transfection with control or two independent SHOX2‐targeting siRNAs. (F) Western blot analysis of SHOX2 and FABP6 isoforms in 769‐P and SN12C cells following SHOX2 knockdown. (G) Schematic workflow of the Co‐IP/MS analysis performed in Caki‐1 cells expressing empty vector (EV) or FABP6L‐3×Flag. (H) Reciprocal exogenous co‐immunoprecipitation assays in HEK293T cells co‐transfected with Flag‐PTEN and either HA‐FABP6L or HA‐FABP6S. (I) Reciprocal endogenous Co‐IP assays in Caki‐1 cells between FABP6L and PTEN. (J) Intensity profile plot of the fluorescence signals for FABP6L (green) and PTEN (red) across a cellular distance (pixels), indicating spatial correlation. (K) Confocal immunofluorescence images in Caki‐1 cells showing cytoplasmic co‐localisation of FABP6L (green) and PTEN (red). Nuclei were stained with DAPI (blue). Scale bar = 20 µm. (L) Subcellular fractionation followed by Western blot analysis of PTEN in Caki‐1 and A‐498 cells expressing empty vector (EV) or FABP6L. FABP6L overexpression reduced PTEN abundance without substantially altering its membrane/cytoplasmic distribution. β‐actin and ATPase were used as cytoplasmic and membrane markers, respectively. (M, N) Western blot analysis showing that FABP6L overexpression (OE) reduces PTEN protein in Caki‐1/A‐498 cells (M), while FABP6L knockdown (sh1, sh2) increases PTEN protein in 769‐P/SN12C cells (N). (O) Cycloheximide (CHX; 50 µg/mL) chase assay in 769‐P and SN12C cells at the indicated time points. Left, representative Western blots; right, quantification of normalised PTEN decay curves. (P) Western blot of Caki‐1 cells treated with the proteasome inhibitor MG132 or the lysosome inhibitor chloroquine (CQ). MG132, but not CQ, rescues the FABP6L‐mediated reduction of PTEN. (Q) Representative immunoblots and densitometric quantification of PTEN ubiquitination in Caki‐1 and HEK293T cells following FABP6L depletion with two independent siRNAs. Quantification of ubiquitin smear intensity is shown below the corresponding blots. (R–T) PTEN re‐expression attenuates FABP6L‐associated tumour growth in a Caki‐1 xenograft model. R, representative images of tumours derived from Caki‐1 cells expressing EV + Ctrl, FABP6L‐OE + Ctrl, or FABP6L‐OE + PTEN‐OE. S, tumour growth curves. T, endpoint tumour weights (*n* = 5). For panel Q, quantitative data are presented as mean ± SD from three independent biological experiments (*n* = 3). Each independent experiment contributed one value per condition.

Given that FABP6L lacks canonical DNA‐binding features and our findings in Result 3 implicated PI3K‐AKT pathway activity, we next investigated whether FABP6L might regulate this pathway through protein interactions. We therefore performed co‐immunoprecipitation followed by mass spectrometry (Co‐IP/MS) in Caki‐1 cells expressing Flag‐tagged FABP6L^31^ (Figure 4G and Extended Data Figure 2M). PTEN emerged as a prominent candidate interactor among proteins associated with PI3K‐AKT signalling (Extended Data Figure 2N). This association was subsequently examined by reciprocal exogenous Co‐IP assays in HEK293T cells co‐transfected with Flag‐tagged PTEN and either HA‐tagged FABP6L or HA‐tagged FABP6S. FABP6L showed a detectable association with PTEN, whereas no comparable association was detected for FABP6S under the same experimental conditions (Figure 4H), supporting the isoform specificity of the FABP6L–PTEN interaction. Endogenous Co‐IP in Caki‐1 cells further supported this association (Figure 4I), while immunofluorescence microscopy showed cytoplasmic co‐localisation of FABP6L and PTEN (Figure 4J and K).

Given the role of PTEN as a negative regulator of PI3K‐AKT signalling, we next examined whether FABP6L affects PTEN abundance or subcellular distribution.^9^ Cell fractionation analysis showed that FABP6L modulation did not substantially alter the membrane/cytoplasmic distribution of PTEN^32^, ^33^ (Figure 4L). In contrast, FABP6L overexpression in Caki‐1 and A‐498 cells reduced total PTEN protein abundance (Figure 4M), whereas FABP6L knockdown in 769‐P and SN12C cells increased PTEN protein levels (Figure 4N). Consistently, FABP6L overexpression, but not FABP6S overexpression, was associated with reduced PTEN abundance in Caki‐1 and A‐498 cells (Extended Data Figure 1Q). RT‐qPCR analysis revealed no significant change in *PTEN* mRNA levels following FABP6L knockdown in 769‐P cells (Extended Data Figure 2O), suggesting that the observed changes in PTEN protein abundance were unlikely to result from altered PTEN transcription. To further assess isoform specificity in the same cellular background, we re‐expressed either FABP6L or FABP6S in FABP6L‐depleted 769‐P cells. Re‐expression of FABP6L reversed the increase in PTEN protein abundance and restored AKT phosphorylation at Thr308 and Ser473, whereas FABP6S re‐expression did not produce a comparable rescue (Extended Data Figure 2P).

We therefore examined whether FABP6L affects PTEN protein stability using cycloheximide (CHX) chase assays.^9^ FABP6L depletion significantly delayed PTEN turnover and prolonged PTEN protein half‐life in both 769‐P and SN12C cells (Figure 4O), supporting a role for FABP6L in promoting PTEN protein degradation. To determine the proteolytic pathway involved, cells were treated with either the proteasome inhibitor MG132 or the lysosome inhibitor chloroquine (CQ). MG132 increased PTEN protein abundance, whereas CQ showed little restorative effect (Figure 4P), supporting the involvement of the ubiquitin‐proteasome system in FABP6L‐associated PTEN turnover. Consistent with these findings, cell‐based ubiquitination assays under MG132 treatment showed reduced PTEN polyubiquitination following FABP6L depletion (Figure 4Q).

To directly determine whether PTEN mediates FABP6L‐dependent AKT–mTORC1 signalling and cell growth in the same cellular background, we performed bidirectional genetic rescue experiments. In 769‐P cells, FABP6L depletion increased PTEN abundance and reduced phosphorylation of AKT, 4EBP1, and p70S6K, whereas concomitant PTEN depletion partially restored AKT–mTORC1 signalling (Extended Data Figure 2R). Consistently, PTEN depletion partially rescued the reductions in cell growth, EdU incorporation, and colony formation induced by FABP6L depletion (Extended Data Figure 2S–U).

Conversely, in Caki‐1 cells, FABP6L overexpression reduced PTEN abundance and enhanced AKT–mTORC1 signalling, whereas PTEN re‐expression attenuated these signalling changes (Extended Data Figure 2R). PTEN re‐expression also reversed the proliferative advantage conferred by FABP6L overexpression, as assessed by CCK‐8, EdU incorporation, and colony formation assays (Extended Data Figure 2V‐X).

We next evaluated the functional contribution of PTEN in vivo using a genetic rescue xenograft model. PTEN re‐expression attenuated the enhanced tumour growth induced by FABP6L overexpression, as reflected by reduced tumour growth and endpoint tumour weight (Figure 4R–T). Together, these bidirectional rescue experiments support a major contribution of PTEN to FABP6L‐mediated AKT–mTORC1 activation and ccRCC growth. Consistent with the rescue experiments, FABP6L modulation produced little detectable effect on AKT phosphorylation or cell proliferation in the PTEN‐null ccRCC cell lines 786‐O and OS‐RC‐2 (Extended Data Figure 3A–T). Similarly, FABP6L knockdown or overexpression had limited effects on tumour growth in PTEN‐null 786‐O xenograft models (Extended Data Figure 3U–Z). Quantitative IHC analysis confirmed increased FABP6L staining in FABP6L‐overexpressing tumours and reduced FABP6L staining in FABP6L‐knockdown tumours. PTEN staining remained low across these groups, and no statistically significant differences were detected in the percentage of Ki‐67‐positive tumour cells or in PTEN and p‐AKT (Thr308) H‐scores following FABP6L overexpression or knockdown (Extended Data Figure 4N–O). Together with the bidirectional rescue experiments, these findings support a major contribution of PTEN to FABP6L‐mediated AKT–mTORC1 activation and ccRCC growth.

To assess the clinical relevance of the SHOX2–FABP6L–PTEN relationship, we analysed paired ccRCC specimens. Western blot analysis showed increased SHOX2 and FABP6L protein abundance and reduced PTEN abundance in tumour tissues compared with matched adjacent normal tissues (Extended Data Figure 4A–D). Correlation analysis revealed a positive association between SHOX2 and FABP6L protein levels (*R* = .671, *p* = .002) and an inverse association between FABP6L and PTEN protein levels (*R* = −.553, *p* = .010), whereas the correlation between SHOX2 and PTEN protein levels was not statistically significant (*R* = −.235, *p* = .318) (Extended Data Figure 4E–G). Immunohistochemical analysis further showed higher SHOX2 and lower PTEN expression in ccRCC tissues (Extended Data Figure 4H). In publicly available clinical datasets, *SHOX2* mRNA expression increased with advancing pathologic T stage, whereas *PTEN* mRNA expression showed the opposite trend (Extended Data Figure 4I and J). Higher SHOX2 expression was also associated with poorer overall survival, whereas higher PTEN expression was associated with more favourable overall survival (Extended Data Figure 4K and L). Together, these clinical observations are consistent with the SHOX2–FABP6L–PTEN relationship identified in our experimental models.

### 3.5 Result 5: FABP6L facilitates PTEN–WWP2 association and promotes WWP2‐associated PTEN ubiquitination

To further investigate the mechanism underlying FABP6L‐associated PTEN ubiquitination, we re‐analysed the FABP6L‐interacting proteins identified by Co‐IP/MS and intersected them with reported PTEN‐targeting E3 ubiquitin ligases. WWP2, a previously reported HECT‐domain E3 ligase for PTEN, emerged as a prominent candidate FABP6L‐associated protein ^10^, ^11^ (Figure 5A). Endogenous Co‐IP in Caki‐1 cells showed that FABP6L‐containing complexes included both PTEN and WWP2 (Figure 5B). Confocal immunofluorescence microscopy further showed cytoplasmic co‐localisation of FABP6L, PTEN, and WWP2 (Figure 5C). In addition, sequential immunoprecipitation in cells expressing tagged proteins supported the coexistence of FABP6L, PTEN, and WWP2 within the same semi‐exogenous protein complex (Extended Data Figure 4M). Together, these observations suggested that FABP6L may facilitate the assembly or stabilisation of PTEN–WWP2‐containing protein complexes.

**FIGURE 5:**
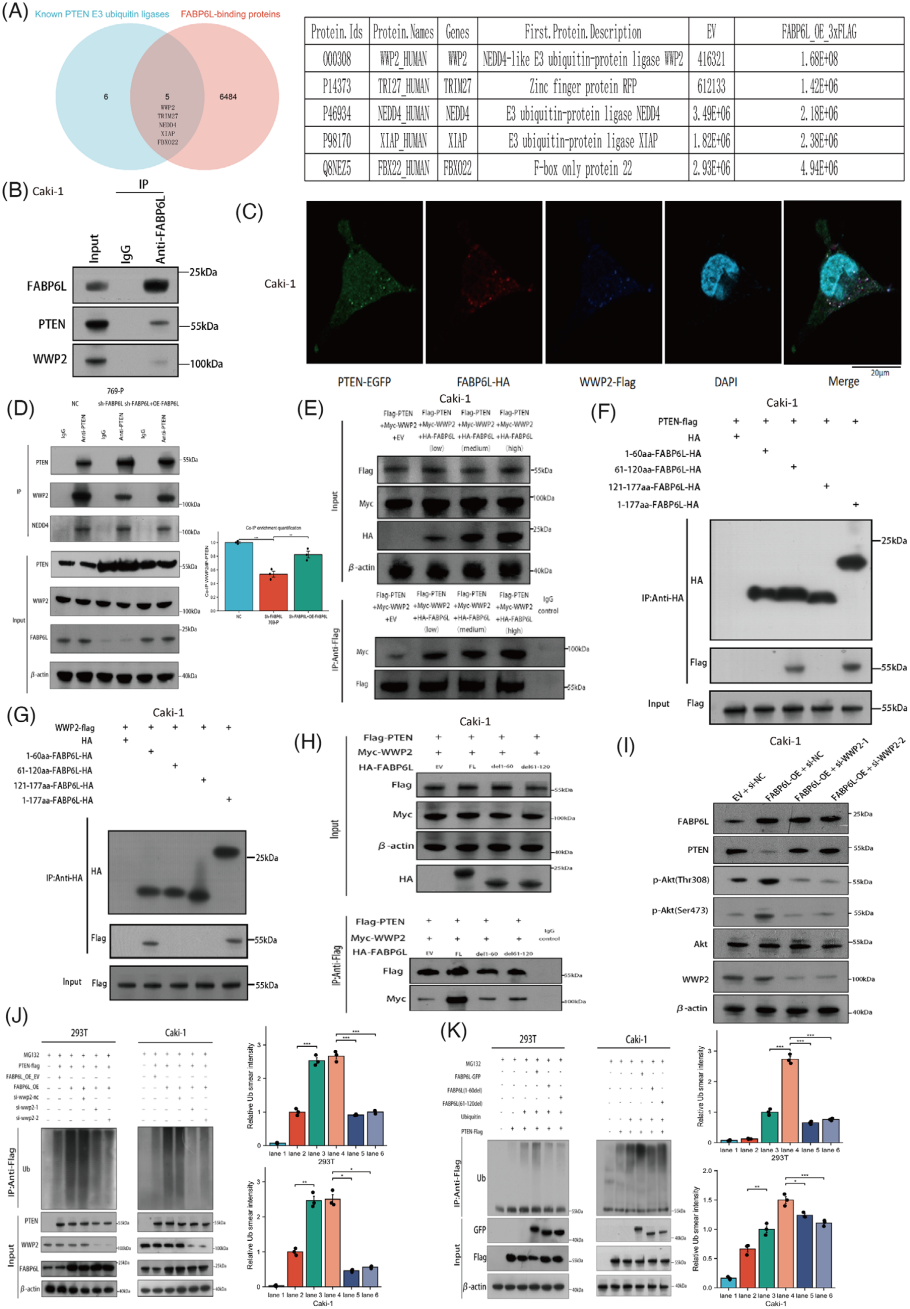
FABP6L facilitates PTEN–WWP2 association and promotes WWP2‐associated PTEN ubiquitination. (A) Intersection of reported PTEN‐targeting E3 ubiquitin ligases with FABP6L‐associated proteins identified by Co‐IP/MS, highlighting WWP2 and other candidate E3 ligases. (B) Endogenous Co‐IP in Caki‐1 cells showing that anti‐FABP6L antibody successfully co‐precipitates both PTEN and the E3 ligase WWP2. (C) Confocal immunofluorescence images showing the cytoplasmic co‐localisation of PTEN‐EGFP (green), FABP6L‐HA (red), and WWP2‐Flag (blue) in ccRCC cells. Scale bar = 20 µm. (D) Representative Co‐IP immunoblots and densitometric quantification of PTEN–WWP2 association in control, FABP6L‐depleted, and FABP6L‐reconstituted 769‐P cells. Co‐immunoprecipitated WWP2 signals were normalised to the corresponding immunoprecipitated PTEN signals and expressed relative to the control group. IgG served as a negative control. NEDD4 was examined in parallel. (E) Co‐IP analysis in Caki‐1 cells co‐expressing Flag‐PTEN and Myc‐WWP2 with increasing amounts of HA‐FABP6L, showing a dose‐dependent increase in WWP2 co‐immunoprecipitation with PTEN. (F) Co‐IP mapping of FABP6L regions contributing to PTEN association using HA‐tagged FABP6L fragments (1–60 aa, 61–120 aa, and 121–177 aa) and full‐length FABP6L. The 61–120‐aa region showed the strongest contribution to PTEN association. (G) Co‐IP mapping of FABP6L regions contributing to WWP2 association using the same truncation constructs. The N‐terminal 1–60‐aa region preferentially contributed to WWP2 association. (H) Co‐IP analysis of PTEN–WWP2 association in the presence of full‐length FABP6L or FABP6L deletion mutants (Δ1–60 and Δ61–120). Full‐length FABP6L enhanced PTEN–WWP2 association, whereas deletion of either region attenuated this effect. (I) Western blot analysis showing that WWP2 depletion restores PTEN abundance and attenuates AKT phosphorylation at Thr308 and Ser473 in FABP6L‐overexpressing Caki‐1 cells. (J) Representative immunoblots and densitometric quantification of cell‐based PTEN ubiquitination assays in HEK293T and Caki‐1 cells showing that depletion of WWP2 with two independent siRNAs reduces FABP6L‐associated PTEN polyubiquitination. Quantification of ubiquitin smear intensity is shown alongside the corresponding blots. (K) Representative immunoblots and densitometric quantification of cell‐based PTEN ubiquitination assays comparing full‐length FABP6L with the Δ1–60 and Δ61–120 deletion mutants in HEK293T and Caki‐1 cells. Deletion of either region attenuated the ability of FABP6L to promote PTEN polyubiquitination. Quantification of ubiquitin smear intensity is shown alongside the corresponding blots. For panels D, J, and K, quantitative data are presented as mean ± SD from three independent biological experiments (*n* = 3). Each independent experiment contributed one value per condition.

We next examined whether FABP6L abundance influenced the association between PTEN and WWP2. FABP6L depletion reduced PTEN–WWP2 co‐immunoprecipitation, whereas re‐expression of FABP6L increased their association towards the control level (Figure 5D). Consistently, immunofluorescence analysis showed reduced spatial co‐localisation between PTEN and WWP2 following FABP6L depletion (Extended Data Figure 5A and B). In a reconstituted system in which PTEN and WWP2 were co‐expressed, increasing amounts of FABP6L progressively enhanced the amount of WWP2 co‐immunoprecipitated with PTEN (Figure 5E). These findings support a role for FABP6L in facilitating PTEN–WWP2 association.

To investigate the regions of FABP6L contributing to these interactions, AlphaFold3 modelling was used as a hypothesis‐generating approach.^34^ The predicted models suggested potential interaction interfaces between FABP6L and WWP2 and between FABP6L and PTEN (Extended Data Figure 5C–F). Co‐IP assays using FABP6L truncation constructs further indicated that the N‐terminal 1–60‐aa region preferentially contributed to WWP2 association, whereas the 61–120‐aa region preferentially contributed to PTEN association (Figure 5F and G). Importantly, in a triple‐expression system containing PTEN and WWP2, full‐length FABP6L enhanced PTEN–WWP2 association, whereas deletion of either residues 1–60 or 61–120 substantially impaired this effect (Figure 5H). These results indicate that both regions contribute to efficient FABP6L‐mediated enhancement of PTEN–WWP2 association. These cellular interaction assays, however, do not establish direct protein–protein contacts at atomic resolution.

We next examined whether WWP2 functionally contributes to FABP6L‐mediated PTEN regulation. FABP6L overexpression reduced PTEN abundance and increased AKT phosphorylation at Thr308 and Ser473, whereas depletion of WWP2 using two independent siRNAs restored PTEN abundance and attenuated AKT phosphorylation in the context of FABP6L overexpression (Figure 5I). Consistently, WWP2 depletion reduced FABP6L‐associated PTEN polyubiquitination (Figure 5J). These findings support a functional contribution of WWP2 to FABP6L‐associated PTEN ubiquitination and downstream AKT activation, while not excluding contributions from other PTEN‐regulating E3 ligases.

We further examined whether the FABP6L regions contributing to PTEN and WWP2 association were required for PTEN ubiquitination. In both HEK293T and Caki‐1 cells, full‐length FABP6L enhanced PTEN polyubiquitination, whereas deletion of either residues 1–60 or 61–120 markedly attenuated this effect (Figure 5K). A complementary ubiquitination assay performed in the presence of exogenous WWP2 showed a similar pattern, with both deletion mutants displaying lower PTEN ubiquitination than full‐length FABP6L (Extended Data Figure 5G). These findings further link the FABP6L regions involved in PTEN/WWP2 association to the regulation of PTEN polyubiquitination.

Isoform‐specific ubiquitination assays provided additional support for this interpretation. Following FABP6L depletion, PTEN polyubiquitination was reduced, and re‐expression of FABP6L restored the ubiquitination signal, whereas FABP6S did not produce a comparable rescue (Extended Data Figure 5H). Consistently, direct comparison of the two isoforms showed greater PTEN polyubiquitination in FABP6L‐expressing cells than in FABP6S‐expressing cells under the same experimental conditions (Extended Data Figure 5I). Together, these results support an isoform‐specific role of FABP6L in promoting PTEN ubiquitination.

Because FABP6 proteins are canonically associated with lipid handling, we additionally examined whether the distinct effects of FABP6L were accompanied by changes in cellular neutral lipid accumulation. Oil Red O staining of Caki‐1 cells expressing empty vector, FABP6S, or FABP6L showed comparable lipid staining among the three groups, and quantitative analysis revealed no significant differences in Oil Red O‐positive area (Extended Data Figure 5J and K). Thus, under the conditions examined, the isoform‐specific effects of FABP6L on PTEN regulation were not accompanied by an obvious alteration in neutral lipid accumulation. Together, these data support a role for FABP6L in facilitating PTEN–WWP2 association and promoting WWP2‐associated PTEN polyubiquitination.

### 3.6 Result 6: FABP6L contributes to reduced everolimus sensitivity through PTEN‐linked AKT/mTORC1 signalling in preclinical ccRCC models

Hyperactivation of the PI3K–AKT signalling pathway is a key contributor to drug resistance across cancer types.^19^, ^35^ In ccRCC, PI3K–AKT–mTOR signalling has been implicated in adaptive resistance to targeted therapy.^4^ Everolimus, an mTOR inhibitor used in ccRCC, exerts its antitumour effects through inhibition of mTOR signalling.^20^ Based on our finding that FABP6L regulates PTEN‐linked AKT/mTORC1 activity, we investigated whether FABP6L modulates the response of ccRCC cells to everolimus. We established an everolimus‐resistant Caki‐1 model (everolimus‐R) through long‐term dose‐escalation selection, and dose–response analysis confirmed reduced everolimus sensitivity compared with parental Caki‐1 cells (Extended Data Figure 5M). RT‐qPCR analysis further showed increased *FABP6L* mRNA expression in everolimus‐R cells compared with parental cells (Figure 6A).

**FIGURE 6:**
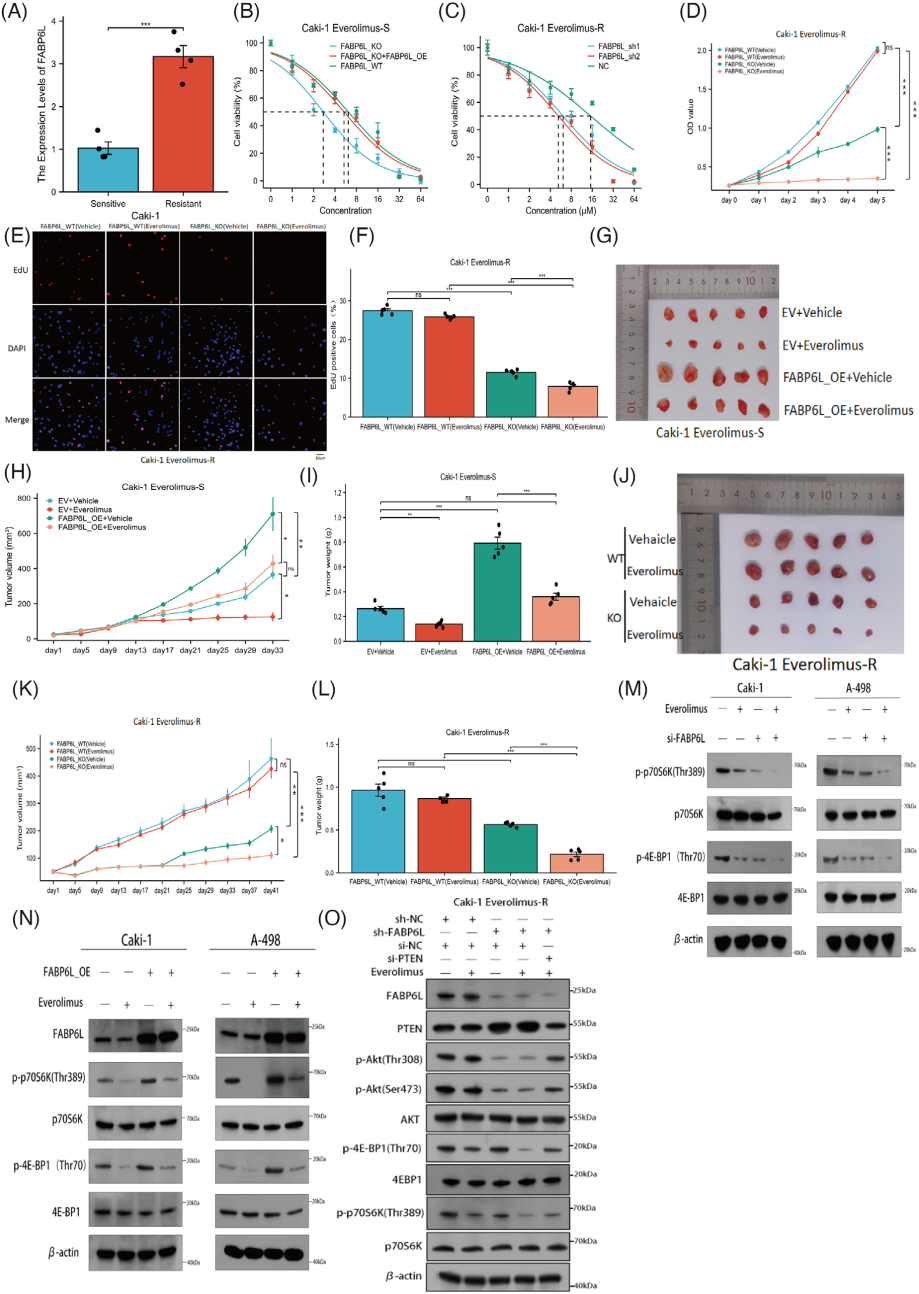
FABP6L contributes to reduced everolimus sensitivity through PTEN‐linked AKT/mTORC1 signalling in preclinical ccRCC models. (A) RT‐qPCR analysis of *FABP6L* mRNA expression in Caki‐1 Everolimus‐S and Caki‐1 Everolimus‐R cells. (B) Everolimus dose–response curves in Caki‐1 Everolimus‐S cells with FABP6L knockout (FABP6L‐KO), FABP6L re‐expression in the knockout background (FABP6L‐KO + rescue), or matched FABP6L‐WT controls. The IC50 values were 2.734 µM (95% CI, 2.573–2.895), 5.352 µM (95% CI, 5.026–5.677), and 6.144 µM (95% CI, 5.766–6.521), respectively. (C) Everolimus dose–response curves in Caki‐1 Everolimus‐R cells expressing FABP6L‐sh1, FABP6L‐sh2, or NC. The IC50 values were 6.096 µM (95% CI, 5.662–6.531), 5.213 µM (95% CI, 4.846–5.581), and 15.18 µM (95% CI, 13.88–16.48), respectively. (D) CCK‐8 proliferation assay in everolimus‐R Caki‐1 cells comparing FABP6L‐WT and FABP6L‐KO cells treated with vehicle or everolimus. (E, F) Representative EdU incorporation images (E) and quantification (F) in everolimus‐R Caki‐1 cells comparing FABP6L‐WT and FABP6L‐KO cells treated with vehicle or everolimus. Scale bar = 50 µm. (G–I) Effect of FABP6L overexpression on the response of Caki‐1 Everolimus‐S xenografts to everolimus (*n* = 5 per group). (G) Representative tumour images; (H) tumour growth curves; (I) endpoint tumour weights. Mice bearing EV or FABP6L‐overexpressing tumours were treated with vehicle or everolimus. (J–L) Effect of FABP6L loss on tumour growth in the Caki‐1 Everolimus‐R xenograft model (*n* = 5 per group). (J) Representative tumour images; (K) tumour growth curves; (L) endpoint tumour weights. FABP6L‐WT and FABP6L‐KO xenografts were treated with vehicle or everolimus. (M) Western blot analysis of mTORC1 downstream signalling in Caki‐1 and A‐498 cells following FABP6L depletion with or without everolimus treatment. Phosphorylation of p70S6K at Thr389 and 4EBP1 at Thr70 was examined. (N) Western blot analysis of p70S6K and 4EBP1 phosphorylation in Caki‐1 and A‐498 cells following FABP6L overexpression with or without everolimus treatment. (O) Western blot analysis of PTEN‐linked AKT/mTORC1 signalling in Caki‐1 Everolimus‐R cells following FABP6L depletion, with or without concomitant PTEN depletion and everolimus treatment.

To investigate the functional impact of FABP6L on everolimus sensitivity, we performed dose–response analyses in both everolimus‐sensitive and everolimus‐resistant Caki‐1 cells. In Caki‐1 Everolimus‐S cells, FABP6L knockout reduced the everolimus IC50 to 2.734 µM (95% CI, 2.573–2.895), compared with 6.144 µM (95% CI, 5.766–6.521) in matched FABP6L‐WT cells. Re‐expression of FABP6L in the knockout background restored the IC50 to 5.352 µM (95% CI, 5.026–5.677), approaching that of FABP6L‐WT cells (Figure 6B). Conversely, in Caki‐1 Everolimus‐R cells, FABP6L knockdown reduced the IC50 from 15.18 µM (95% CI, 13.88–16.48) in control cells to 6.096 µM (95% CI, 5.662–6.531) and 5.213 µM (95% CI, 4.846–5.581) with two independent FABP6L‐targeting shRNAs, respectively (Figure 6C).

Using everolimus‐R Caki‐1 cells, we further examined the effect of FABP6L depletion on cell proliferation during everolimus treatment. FABP6L depletion reduced cell growth, and everolimus exerted a greater growth‐inhibitory effect in FABP6L‐depleted cells than in control cells, as assessed by CCK‐8 assays (Figure 6D). Consistently, EdU incorporation was reduced following FABP6L depletion and was further decreased under everolimus treatment (Figure 6E and F). These results indicate that loss of FABP6L enhances the growth‐inhibitory response of everolimus‐resistant ccRCC cells to everolimus.

To validate these findings in vivo, we first established xenografts using parental Caki‐1 cells expressing either empty vector or FABP6L. Under vehicle treatment, FABP6L‐overexpressing tumours exhibited increased tumour growth and endpoint tumour weight compared with empty‐vector controls. Everolimus suppressed tumour growth in both groups; however, FABP6L‐overexpressing tumours remained larger and heavier than empty‐vector tumours under everolimus treatment (Figure 6G–I). These findings indicate that FABP6L overexpression reduces the antitumour response to everolimus in parental Caki‐1 xenografts.

We further evaluated FABP6L loss in the everolimus‐R Caki‐1 xenograft model. FABP6L knockout reduced tumour growth compared with WT tumours, and FABP6L‐KO tumours treated with everolimus showed the lowest tumour burden among the four experimental groups (Figure 6J–L). Immunohistochemical analysis of xenograft tissues further showed increased PTEN staining together with reduced Ki‐67, phospho‐AKT, and phospho‐4EBP1 staining following FABP6L loss, particularly under everolimus treatment (Extended Data Figure 5L). These observations further support a role for FABP6L in modulating everolimus response in vivo.

At the cellular signalling level, Western blot analysis showed that FABP6L depletion reduced phosphorylation of the mTORC1 downstream effectors p70S6K and 4EBP1 in Caki‐1 and A‐498 cells and enhanced their suppression during everolimus treatment (Figure 6M). Conversely, FABP6L overexpression increased basal phosphorylation of p70S6K and 4EBP1 and partially maintained their phosphorylation during everolimus exposure (Figure 6N), consistent with a role for FABP6L in sustaining mTORC1 pathway activity.

To further examine whether PTEN contributes to the signalling changes associated with FABP6L depletion, we performed a genetic rescue experiment in everolimus‐R Caki‐1 cells. FABP6L depletion increased PTEN abundance and reduced AKT phosphorylation at Thr308 and Ser473, accompanied by decreased phosphorylation of 4EBP1 and p70S6K. These effects were further evident under everolimus treatment. Importantly, concomitant PTEN depletion partially restored AKT phosphorylation and downstream mTORC1 signalling in FABP6L‐depleted cells treated with everolimus (Figure 6O). Together, these findings indicate that FABP6L contributes to reduced everolimus sensitivity in preclinical ccRCC models, at least in part through PTEN‐linked AKT/mTORC1 signalling.

## 4 DISCUSSION

Although targeted therapies and immunotherapies have substantially improved outcomes for patients with advanced clear cell renal cell carcinoma (ccRCC), therapeutic resistance and tumour recurrence remain major clinical challenges.^36^ Recent studies have linked ccRCC pathogenesis to lipid metabolic reprogramming and aberrant activation of the PI3K‐AKT‐mTOR signalling pathway, with members of the fatty acid binding protein (FABP) family contributing to these metabolic and signalling alterations.^37^, ^38^ However, previous studies largely evaluated FABP6 without distinguishing its individual isoforms.^17^ Here, through isoform‐resolved analyses and functional experiments, we identify FABP6L as the predominant tumour‐upregulated FABP6 isoform in ccRCC. Mechanistically, our data support a model in which FABP6L facilitates PTEN–WWP2 association and promotes WWP2‐associated PTEN polyubiquitination and proteasomal degradation, thereby enhancing AKT/mTOR signalling and modulating the response to everolimus. Collectively, these findings identify FABP6L as an isoform‐specific regulator of PTEN‐linked AKT/mTOR signalling and suggest a role for FABP6L in modulating the response to mTOR inhibition in preclinical ccRCC models.

As a core member of the FABP family, FABP6 is classically involved in bile acid transport and lipid homeostasis.^23^ Human FABP6 undergoes alternative splicing to generate two major transcripts encoding a short isoform (128 amino acids) and a long isoform (177 amino acids).^12^ While FABP6 has been implicated in various cancers, including esophageal cancer, bladder cancer, and glioblastoma, prior studies generally did not distinguish between these two isoforms, potentially obscuring isoform‐specific functions.^14^, ^18^ In this study, we demonstrate that FABP6L is the dominant isoform in ccRCC. Using isoform‐specific RT‐qPCR and FABP6L‐specific protein analyses, we confirmed increased FABP6L expression in clinical ccRCC specimens. Publicly available datasets further showed that total FABP6 expression was associated with pathologic T stage and grade; however, these datasets did not resolve individual FABP6 isoforms. Public single‐cell RNA‐seq analysis further showed that total FABP6 signal was enriched in the malignant epithelial compartment; however, these datasets did not resolve FABP6L from FABP6S. Functional assays further showed that FABP6L promotes ccRCC cell proliferation and tumour growth, whereas parallel FABP6S controls supported an isoform‐specific effect of FABP6L. Because FABP6 is classically associated with lipid handling, we also examined neutral lipid accumulation. Oil Red O staining revealed no significant differences among empty‐vector, FABP6S‐overexpressing, and FABP6L‐overexpressing cells, indicating that the isoform‐specific effects of FABP6L observed here were not accompanied by an obvious change in neutral lipid accumulation under the conditions examined. These findings do not, however, exclude a role for FABP6L in other aspects of lipid metabolism or directly establish whether FABP6L retains intrinsic fatty acid‐binding activity. Nevertheless, its expression dynamics in metastatic lesions and potential role in metastasis warrant further investigation.

Low expression of PTEN is a well‐established feature associated with tumour progression and drug resistance.^39^, ^40^ Known mechanisms contributing to PTEN downregulation include genomic deletions, transcriptional repression, and post‐translational modifications such as ubiquitination mediated by E3 ligases like WWP1 and NEDD4‐1.^32^, ^41^ However, the precise mechanisms governing PTEN stability in ccRCC remain incompletely defined.^42^, ^43^ Our findings support a post‐translational regulatory mechanism in which FABP6L facilitates the association between PTEN and WWP2 and promotes WWP2‐associated PTEN ubiquitination. Domain‐mapping experiments suggest that the FABP6L 1–60‐aa region preferentially contributes to WWP2 association, whereas the 61–120‐aa region contributes to PTEN association. Deletion of either region impaired PTEN–WWP2 association and reduced FABP6L‐associated PTEN ubiquitination. WWP2 depletion further attenuated FABP6L‐associated PTEN ubiquitination and downstream AKT signalling, supporting a functional contribution of WWP2 to this process without excluding the involvement of additional E3 ligases. However, these cellular interaction and domain‐mapping experiments do not establish direct protein–protein contacts at atomic resolution. AlphaFold3 modelling should therefore be considered hypothesis‐generating, and purified‐protein reconstitution together with high‐resolution structural studies will be required to define the precise interaction interfaces and distinguish direct from complex‐mediated contacts.

Clinical resistance to PI3K‐AKT‐mTOR pathway inhibition remains a formidable obstacle in targeted cancer therapy.^44^ Everolimus, an mTORC1 specific inhibitor, exhibits heterogeneous efficacy in advanced RCC, with most patients eventually developing acquired resistance.^45^ Well‐documented mechanisms of resistance include compensatory PI3K‐AKT reactivation through upstream receptor tyrosine kinases, secondary mutations in mTOR or its downstream effectors, and epigenetic alterations.^46^, ^47^ Activation of the PI3K pathway is a common feature underlying resistance.^4^ Our findings suggest that the FABP6L–WWP2–PTEN axis may contribute to reduced everolimus sensitivity by promoting PTEN loss and sustaining AKT/mTOR signalling. Consistent with this model, FABP6L depletion increased everolimus sensitivity in resistant Caki‐1 cells, whereas FABP6L overexpression reduced the antitumour response to everolimus in parental Caki‐1 xenografts. In the everolimus‐resistant xenograft model, FABP6L loss further reduced tumour growth and was associated with increased PTEN abundance and decreased AKT/mTORC1 pathway activity. Moreover, PTEN depletion in FABP6L‐depleted resistant cells partially restored AKT phosphorylation and downstream mTORC1 signalling, supporting a contribution of PTEN restoration to the signalling effects of FABP6L depletion. Several limitations should be considered. First, the everolimus‐resistance experiments were performed primarily using a single experimentally generated resistant Caki‐1 model, and validation in additional independently established resistant ccRCC models will be required. Second, clinically annotated cohorts of everolimus‐treated patients were not available to directly evaluate whether FABP6L expression is associated with treatment response. Therefore, the present findings support a preclinical role for FABP6L in modulating everolimus sensitivity but do not establish FABP6L as a clinical biomarker of everolimus resistance. Furthermore, given that historical studies overlooked FABP6 isoform diversity, characterising the broader implications of the FABP6L–PTEN axis in drug responses across other malignancies represents a compelling direction for future research.^17^, ^23^

In conclusion, this study identifies FABP6L as an isoform‐specific regulator of PTEN stability and AKT/mTOR signalling in ccRCC. Our findings support a model in which FABP6L facilitates PTEN–WWP2 association and WWP2‐associated PTEN polyubiquitination and degradation, thereby promoting tumour growth and contributing to reduced everolimus sensitivity in preclinical models (Figure 7).

**FIGURE 7:**
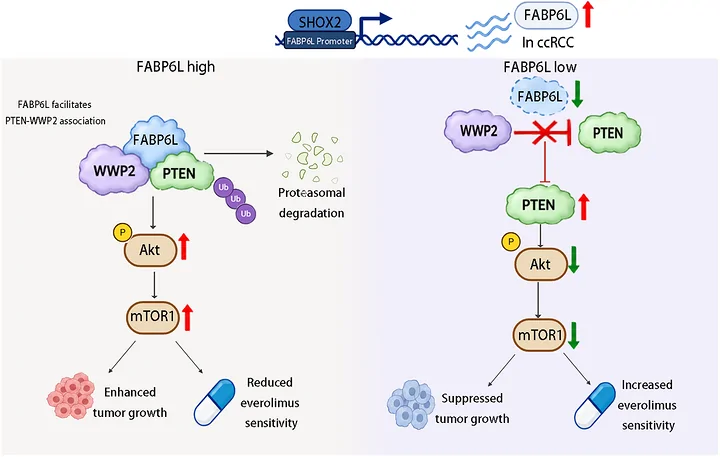
Proposed mechanistic model of FABP6L‐mediated PTEN regulation and everolimus sensitivity in ccRCC. Left panel (FABP6L high): SHOX2 contributes to transcriptional upregulation of FABP6L in ccRCC. Elevated FABP6L facilitates PTEN–WWP2 association and promotes WWP2‐associated PTEN polyubiquitination and proteasomal degradation. Reduced PTEN abundance is associated with enhanced AKT/mTORC1 signalling, increased tumour growth, and reduced everolimus sensitivity. Right panel (FABP6L low): Reduced FABP6L expression is associated with decreased PTEN–WWP2 association and reduced PTEN degradation, resulting in increased PTEN abundance. Increased PTEN abundance is associated with reduced AKT/mTORC1 signalling, suppressed tumour growth, and increased everolimus sensitivity.

## AUTHOR CONTRIBUTIONS

Haoming Yin, Zijie Wang, Liyuan Ge, and Wei Liu were involved in the study conceptualisation, data curation, formal analysis, investigation, and writing of the original draft. Fan Shu and Ye Yan participated in data acquisition, methodology development, and manuscript review and editing. Hui Liang and Shudong Zhang provided supervision, project administration, funding acquisition, and critical revision of the manuscript. All authors have read and approved the final version of the manuscript.

## CONFLICT OF INTEREST STATEMENT

The authors declare that they have no conflict of interest.

## ETHICS STATEMENT

The use of clinical samples in this study was approved by the Medical Ethics Committee of Peking University Third Hospital (Nos. [M2017147] and [IRB0000‐M20260071]), and written informed consent was obtained from all patients in accordance with the Declaration of Helsinki. All animal experiments were approved by the Institutional Animal Care and Use Committee (IACUC) of Peking University Health Science Center (No. DLASBD0141) and conducted following the relevant guidelines for animal welfare.

## Supporting information



SUPPORTING INFORMATION

SUPPORTING INFORMATION

SUPPORTING INFORMATION

SUPPORTING INFORMATION

SUPPORTING INFORMATION

SUPPORTING INFORMATION

SUPPORTING INFORMATION

SUPPORTING INFORMATION

SUPPORTING INFORMATION

SUPPORTING INFORMATION

SUPPORTING INFORMATION

## Data Availability

The mass spectrometry proteomics data have been deposited in the ProteomeXchange Consortium via the iProX partner repository under dataset identifier PXD077055 and iProX project identifier IPX0016559000. Other raw datasets generated in this study are available from the corresponding authors upon reasonable request.
