# Supplementary material for: The long isoform of FABP6 facilitates WWP2‐mediated PTEN ubiquitination to activate AKT/mTOR signalling and contributes to reduced everolimus sensitivity in clear cell renal cell carcinoma
Source: Clin Transl Med. 2026 Oct 24;16(10):e70880. doi: 10.1002/ctm2.70880 (PMC13695829; doi:10.1002/ctm2.70880)

SN12C cells STR report

Method: An appropriate amount of "SN12C" cells (Cell number PC-H2026030523,1×10^6) were used TIANamp Genomic DNA Kit to extract DNA, 20 STR loci and gender identification loci were amplified by Microreader™21 ID System, PCR product detection was performed by GenReader 7010 genetic analyzer, detection results were analyzed by GeneMapper Software6 (Applied Biosystems), and compared with Cellosaurus databases.

Experimental result:

1. The results of negative and positive control were correct.
2. The genotyping results of STR locus of "SN12C" cell line is shown in the following table.

Conclusion:

1. The STR profiles of the genomic DNA of the "SN12C" cell line were clear after amplification, and the result of genotyping is good.
2. The results of STR typing showed that no cross contamination of human cell was found in the cell line of "SN12C" cell line.
3. The STR typing data of this cell line matched the STR data of the SN12C cell line in the Cellosaurus database, with a matching rate of 100.00%.

Appendix II: The genotyping results of STR locus of "SN12C" (Cell number PC-H2026030523) cell line.

Applied Biosystems
GeneMapper Software 6

20260305

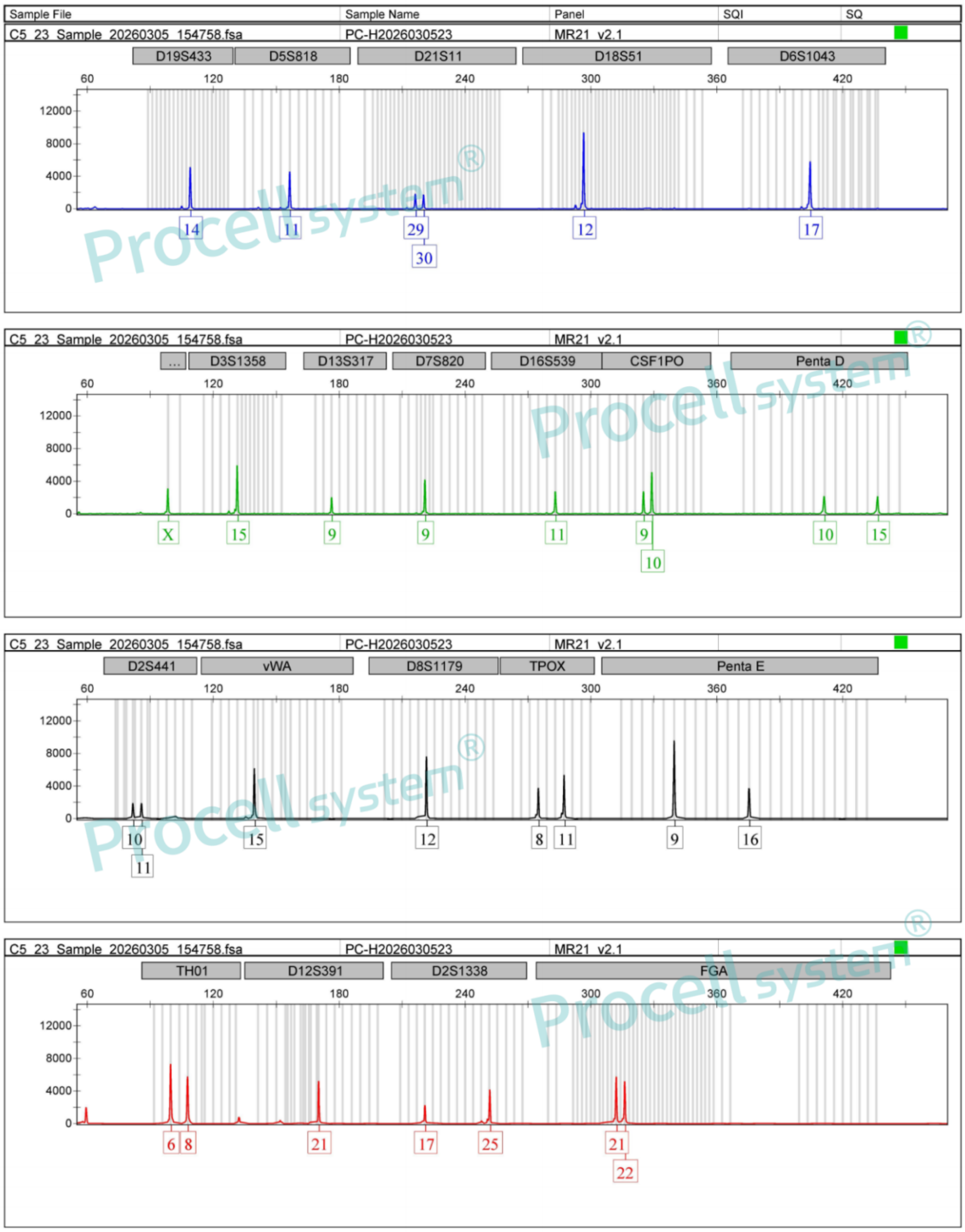

Fri Mar 06,2026 09:19AM, CST   Printed by: gm   Page 1 of 1

Appendix I: The genotyping results of STR locus of "SN12C" cell line.

STR Loci	Sample: PC-H2026030523	Database：SN12C
Amelogenin	X	X
CSF1PO	9,10	9,10
D2S1338	17,25	17,25
D3S1358	15	15
D5S818	11	11
D7S820	9	9
D8S1179	12	12
D13S317	9	9
D16S539	11	11
D18S51	12	12
D19S433	14	14
D21S11	29,30	29,30
FGA	21,22	21,22
PentaD	10,15	
PentaE	9,16	
TH01	6,8	6,8
TPOX	8,11	8,11
vWA	15	15
D6S1043	17	
D12S391	21	
D2S441	10,11	
The Cellosaurus database has a matching rate of 100.00%, The number of matched bits is 15 (https://www.cellosaurus.org/index.html)		

Note:

1. According to the cell STR identification standard established by the International Cell Line Authentication Committee (ICLAC), when the matching degree of cell lines is ≥80%, they are considered to be correlated, that is, derived from common ancestral cells; The matching degree is between 55% and 80%, and the correlation needs to be further verified. Less than 55% indicates no correlation between the two.
2. The effective peak of the map was the real PCR band; Small peaks and nonspecific bands were ignored in the calculation.
3. The default Cellosaurus STR data comparing the results, the data sources include ATCC, DSMZ, JCRB cell library and documents and records, such as https://www.cellosaurus.org/index.html database entry.

细胞遗传质量鉴定检测

Cell Line Authentication Service

一、细胞名称：293T

二、检测方法：用 Axygen 的基因组抽提试剂盒提取 DNA，采用 21- STR 扩增方案扩增，在 ABI 3730XL 型遗传分析仪上对 STR 位点和性别基因 Amelogenin 进行检测。

三、样本基因分型结果：

细胞的 STR 位点和 Amelogenin 位点的基因分型结果						
Loci	送检细胞 STR 信息			细胞库细胞 STR 信息		
	送检细胞名：293T			细胞库细胞名：HEK293T		
	Allele1	Allele2	Allele3	Allele1	Allele2	Allele3
D5S818	8	9		8	9	
D13S317	12	12		12	12	
D7S820	11	11		11	11	
D16S539	9	13		9	13	
VWA	16	19		16	19	
TH01	7	9.3		7	9.3	
AMEL	X	X		X	X	
TPOX	11	11		11	11	
CSF1PO	11	12		11	12	
D12S391	19	21				
FGA	23	23				
D2S1338	19	19				
D21S11	28	30.2				
D18S51	17	18				
D8S1179	12	14				
D3S1358	15	17				
D6S1043	11	11				

PENTAE	7	15				
D19S433	18	18				
PENTAD	9	10				
D1S1656	15	17.3				

四、结论：**293T** 细胞 STR 分型结果与 **DSMZ** 数据库 **293T** 细胞系基因型一致，细胞号对应 **CVCL_0063Best**，STR 分型结果**完全匹配**。本次检测在该细胞系中**没有发现多等位基因**。

五、附图

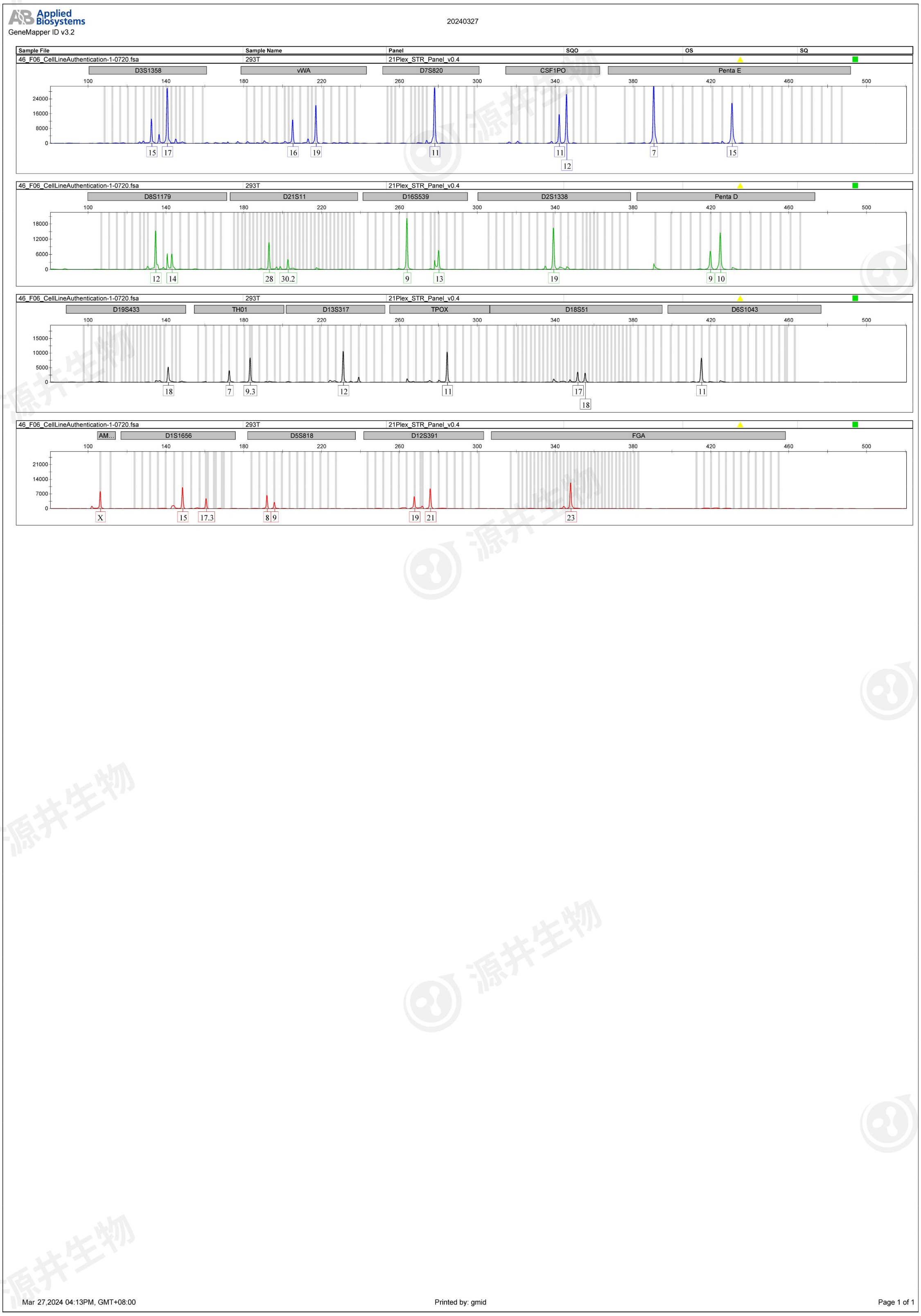
Applied Biosystems
GeneMapper ID v3.2
20240327
Sample File
Sample Name
Panel
SQO
OS
SQ
46_F06_CellLineAuthentication-1-0720.fsa
293T
21Plex_STR_Panel_v0.4
D3S1358
vWA
D7S820
CSF1PO
Penta E
15 17
16 19
11
11 12
7
15
D8S1179
D21S11
D16S539
D2S1338
Penta D
12 14
28 30.2
9 13
19
9 10
D19S433
TH01
D13S317
TPOX
D18S51
D6S1043
18
7 9.3
12
11
17 18
11
AM...
D1S1656
D5S818
D12S391
FGA
X
15 17.3
8 9
19 21
23
Mar 27,2024 04:13PM, GMT+08:00
Printed by: gmid
Page 1 of 1

细胞遗传质量鉴定检测

Cell Line Authentication Service

一、细胞名称：769-P

二、检测方法：用 Axygen 的基因组抽提试剂盒提取 DNA，采用 21- STR 扩增方案扩增，在 ABI 3730XL 型遗传分析仪上对 STR 位点和性别基因 Amelogenin 进行检测。

三、样本基因分型结果：

细胞的 STR 位点和 Amelogenin 位点的基因分型结果						
Loci	送检细胞 STR 信息			细胞库细胞 STR 信息		
	送检细胞名：STR24103112			细胞库细胞名：769-P		
	Allele1	Allele2	Allele3	Allele1	Allele2	Allele3
D5S818	12	12		12	12	
D13S317	10	14		10	14	
D7S820	10	11		10	11	
D16S539	9	13		9	13	
VWA	18	18		18	18	
TH01	6	9.3		6	9.3	
AMEL	X	X		X	X	
TPOX	8	11		8	11	
CSF1PO	11	12		11	12	
D12S391	18	18				
FGA	20	22				
D2S1338	18	18				
D21S11	28	29	30.0			
D18S51	14	17				
D8S1179	12	16				
D3S1358	16	16				
D6S1043	11	14				

PENTAE	7	18				
D19S433	14	15				
PENTAD	12	16				
D1S1656	16	17.3				

四、结论：**769-P** 细胞 STR 分型结果与 **DSMZ** 数据库 **769-P** 细胞系基因型一致，细胞号对应 **CRL-1933**，STR 分型结果**基本匹配**。本次检测在该细胞系中**没有发现多等位基因**。

五、附图

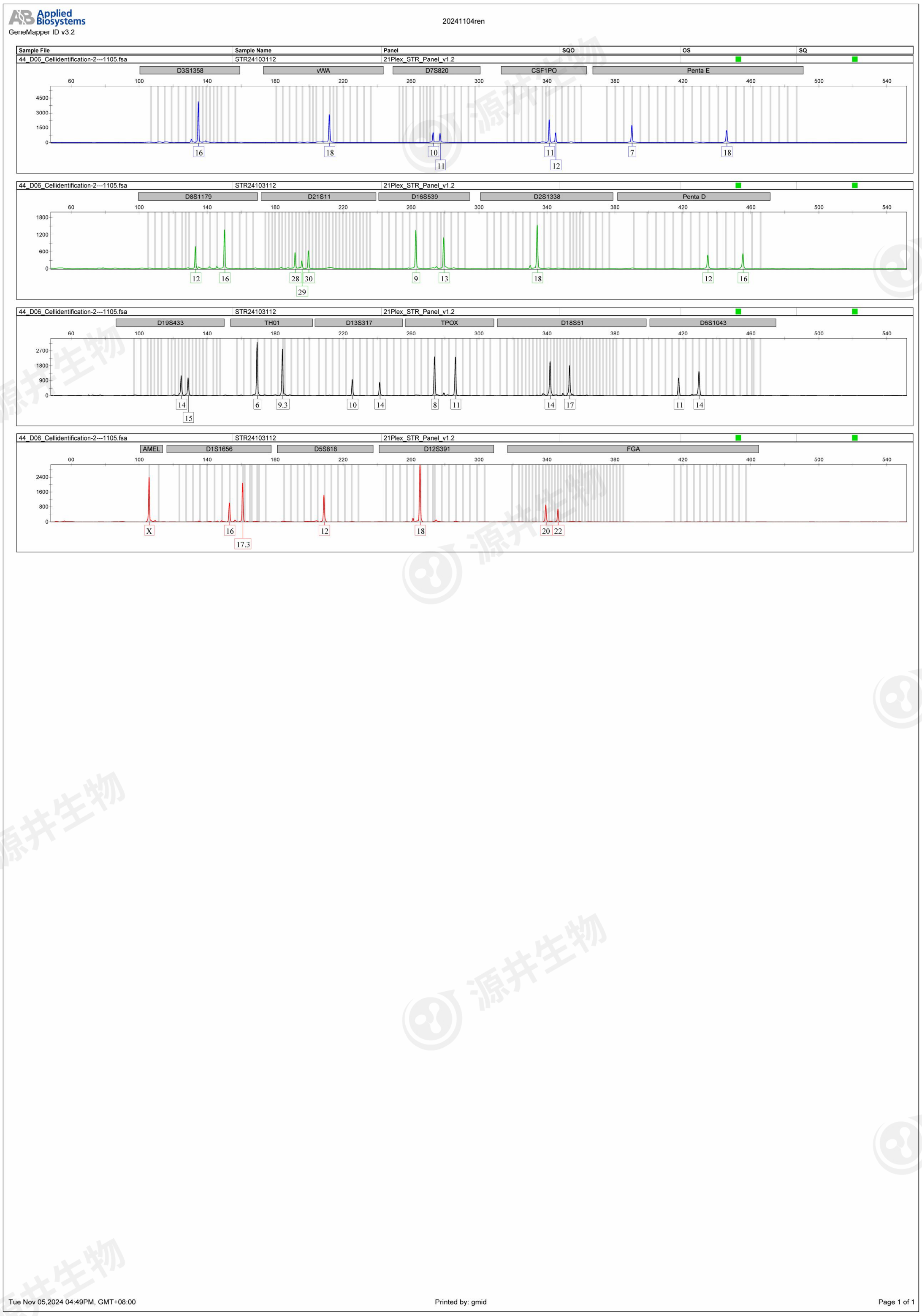
Applied Biosystems
GeneMapper ID v3.2
20241104ren
Sample File
Sample Name
Panel
SQO
OS
SQ
44_D06_Cellidentification-2---1105.fsa
STR24103112
21Plex_STR_Panel_v1.2
D3S1358
vWA
D7S820
CSF1PO
Penta E
16
18
10
11
11
12
7
18
D8S1179
D21S11
D16S539
D2S1338
Penta D
12
16
28
29
30
9
13
18
12
16
D19S433
TH01
D13S317
TPOX
D18S51
D6S1043
14
15
6
9.3
10
14
8
11
14
17
11
14
AMEL
D1S1656
D5S818
D12S391
FGA
X
16
17.3
12
18
20
22
Tue Nov 05,2024 04:49PM, GMT+08:00
Printed by: gmid
Page 1 of 1

细胞遗传质量鉴定检测

Cell Line Authentication Service

一、细胞名称：786-0

二、检测方法：用 Axygen 的基因组抽提试剂盒提取 DNA，采用 21- STR 扩增方案扩增，在 ABI 3730XL 型遗传分析仪上对 STR 位点和性别基因 Amelogenin 进行检测。

三、样本基因分型结果：

细胞的 STR 位点和 Amelogenin 位点的基因分型结果						
Loci	送检细胞 STR 信息			细胞库细胞 STR 信息		
	送检细胞名：786-0			细胞库细胞名：786-O [786-0]		
	Allele1	Allele2	Allele3	Allele1	Allele2	Allele3
D5S818	9	9		9	9	
D13S317	8	8		8	8	
D7S820	11	12		11	12	
D16S539	12	12		12	12	
VWA	15	17		15	17	
TH01	6	9.3		6	9.3	
AMEL	X	Y		X	Y	
TPOX	8	11		8	11	
CSF1PO	10	10		10	10	
D12S391	17	18				
FGA	24	24				
D2S1338	17	18				
D21S11	29	30				
D18S51	13	14				
D8S1179	13	13				
D3S1358	16	16				
D6S1043	11	11				

PENTAE	7	16				
D19S433	14	15				
PENTAD	9	12				
D1S1656	11	12				

四、结论：**786-0** 细胞 STR 分型结果与 **DSMZ** 数据库 **786-0** 细胞系基因型一致，细胞号对应 **CRL-1932**，STR 分型结果**完全匹配**。本次检测在该细胞系中**没有发现多等位基因**。

五、附图

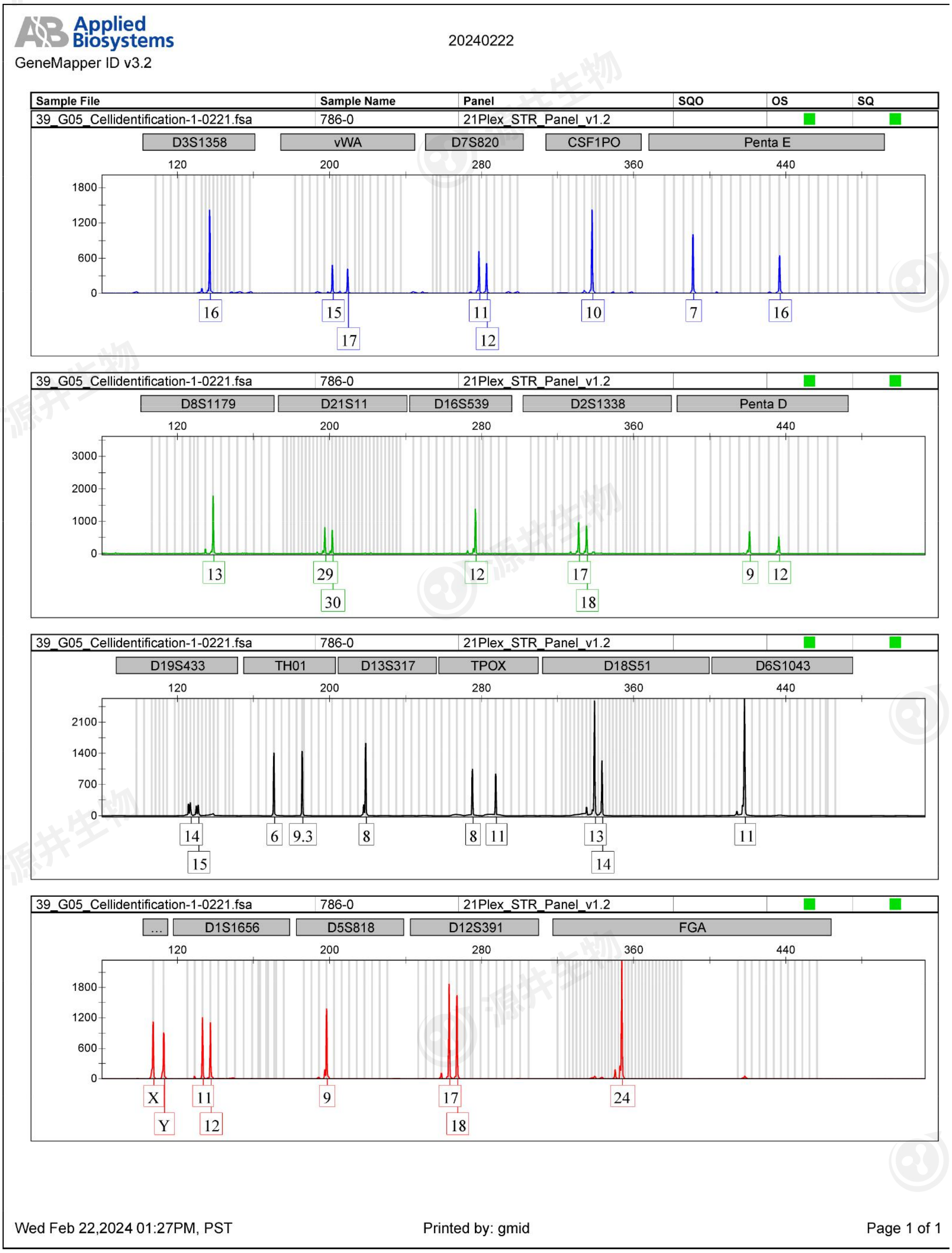
Applied Biosystems
20240222
GeneMapper ID v3.2
Sample File
Sample Name
Panel
SQO
OS
SQ
39_G05_Cellidentification-1-0221.fsa
786-0
21Plex_STR_Panel_v1.2
D3S1358
vWA
D7S820
CSF1PO
Penta E
16
15
17
11
12
10
7
16
D8S1179
D21S11
D16S539
D2S1338
Penta D
13
29
30
12
17
18
9
12
D19S433
TH01
D13S317
TPOX
D18S51
D6S1043
14
15
6
9.3
8
8
11
13
14
11
D1S1656
D5S818
D12S391
FGA
X
Y
11
12
9
17
18
24
Wed Feb 22,2024 01:27PM, PST
Printed by: gmid
Page 1 of 1

细胞遗传质量鉴定检测

Cell Line Authentication Service

一、细胞名称：A-498

二、检测方法：用 Axygen 的基因组抽提试剂盒提取 DNA，采用 21- STR 扩增方案扩增，在 ABI 3730XL 型遗传分析仪上对 STR 位点和性别基因 Amelogenin 进行检测。

三、样本基因分型结果：

细胞的 STR 位点和 Amelogenin 位点的基因分型结果						
Loci	送检细胞 STR 信息			细胞库细胞 STR 信息		
	送检细胞名：STR24091210			细胞库细胞名：A-498		
	Allele1	Allele2	Allele3	Allele1	Allele2	Allele3
D5S818	11	13		11	13	
D13S317	12	12		12	12	
D7S820	10	11		10	11	
D16S539	12	12		12	12	
VWA	18	18		18	18	
TH01	6	9.3		6	9.3	
AMEL	X	X		X	X	
TPOX	8	11		8	11	
CSF1PO	11	12		11	12	
D12S391	21	21				
FGA	18	20				
D2S1338	26	26				
D21S11	28	32				
D18S51	17	17				
D8S1179	13	15				
D3S1358	15	15				
D6S1043	11	11				

PENTAE	10	14				
D19S433	12	12				
PENTAD	9	14				
D1S1656	14	17.3				

四、结论：**A-498** 细胞 STR 分型结果与 **DSMZ** 数据库 **A-498** 细胞系基因型一致，细胞号对应 **ACC-CRL-7908**，STR 分型结果**完全匹配**。本次检测在该细胞系中**没有发现多等位基因**。

五、附图

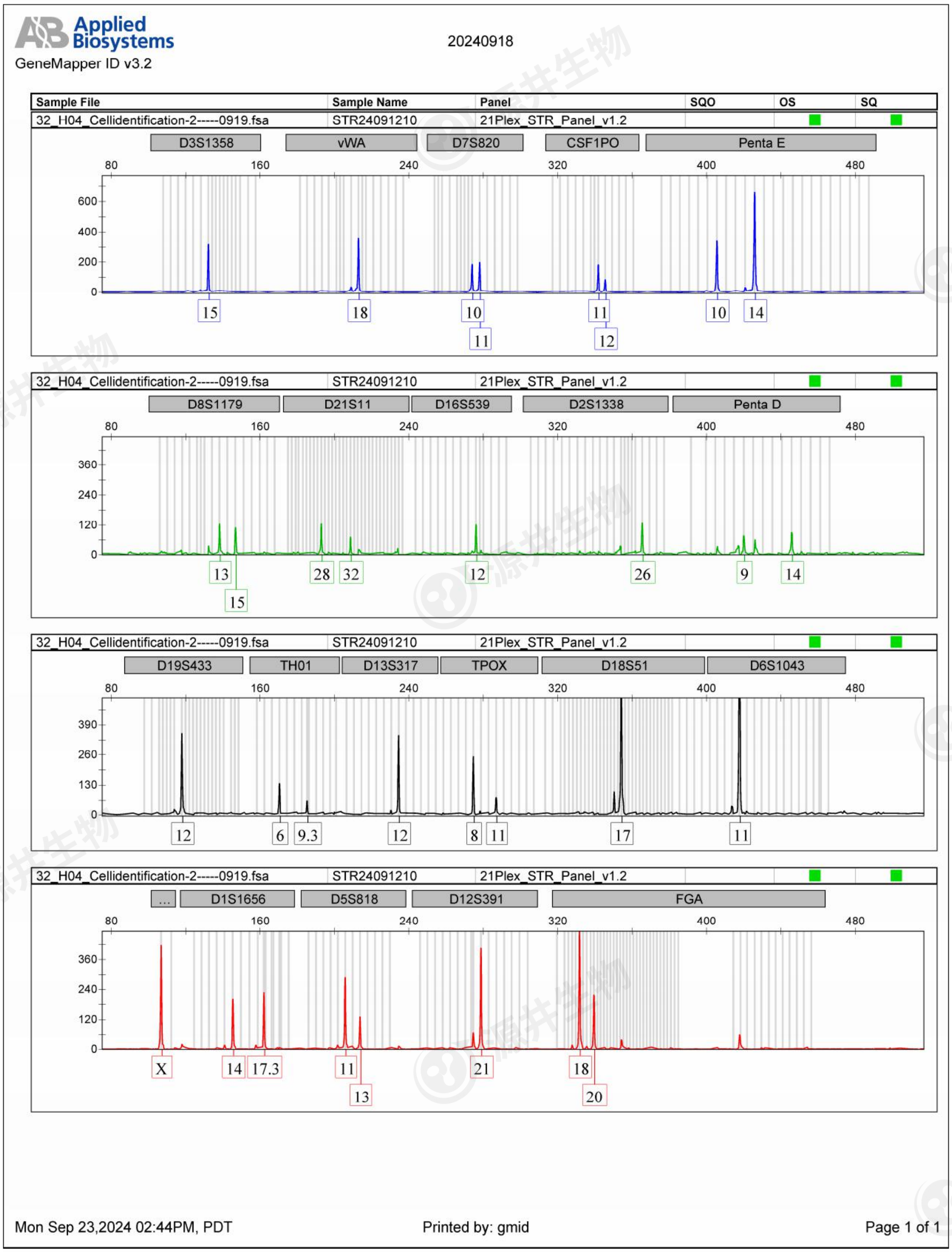
Applied Biosystems
GeneMapper ID v3.2
20240918
Sample File
Sample Name
Panel
SQO
OS
SQ
32_H04_Cellidentification-2-----0919.fsa
STR24091210
21Plex_STR_Panel_v1.2
D3S1358
vWA
D7S820
CSF1PO
Penta E
15
18
10
11
11
12
10
14
D8S1179
D21S11
D16S539
D2S1338
Penta D
13
15
28
32
12
26
9
14
D19S433
TH01
D13S317
TPOX
D18S51
D6S1043
12
6
9.3
12
8
11
17
11
D1S1656
D5S818
D12S391
FGA
X
14
17.3
11
13
21
18
20
Mon Sep 23,2024 02:44PM, PDT
Printed by: gmid
Page 1 of 1

细胞遗传质量鉴定检测

Cell Line Authentication Service

一、细胞名称：Caki-1

二、检测方法：用 Axygen 的基因组抽提试剂盒提取 DNA，采用 21- STR 扩增方案扩增，在 ABI 3730XL 型遗传分析仪上对 STR 位点和性别基因 Amelogenin 进行检测。

三、样本基因分型结果：

细胞的 STR 位点和 Amelogenin 位点的基因分型结果						
Loci	送检细胞 STR 信息			细胞库细胞 STR 信息		
	送检细胞名：STR24103102			细胞库细胞名：CAKI-1		
	Allele1	Allele2	Allele3	Allele1	Allele2	Allele3
D5S818	11	12		11	12	
D13S317	11	12		11	12	
D7S820	8	12		8	12	
D16S539	12	12		12	12	
VWA	15	17		15	17	
TH01	6	8		6	8	
AMEL	X	X		X	X	
TPOX	8	11		8	11	
CSF1PO	10	11		10	11	
D12S391	15	17				
FGA	26	26				
D2S1338	17	19				
D21S11	28	30				
D18S51	14	14				
D8S1179	12	14				
D3S1358	17	17				
D6S1043	11	12				

PENTAE	11	22				
D19S433	14	14				
PENTAD	11	12				
D1S1656	15	20.3				

四、结论：**Caki-1** 细胞 STR 分型结果与 **DSMZ** 数据库 **Caki-1** 细胞系基因型一致，细胞号对应 **ACC-HTB-46**，STR 分型结果**完全匹配**。本次检测在该细胞系中**没有发现多等位基因**。

五、附图

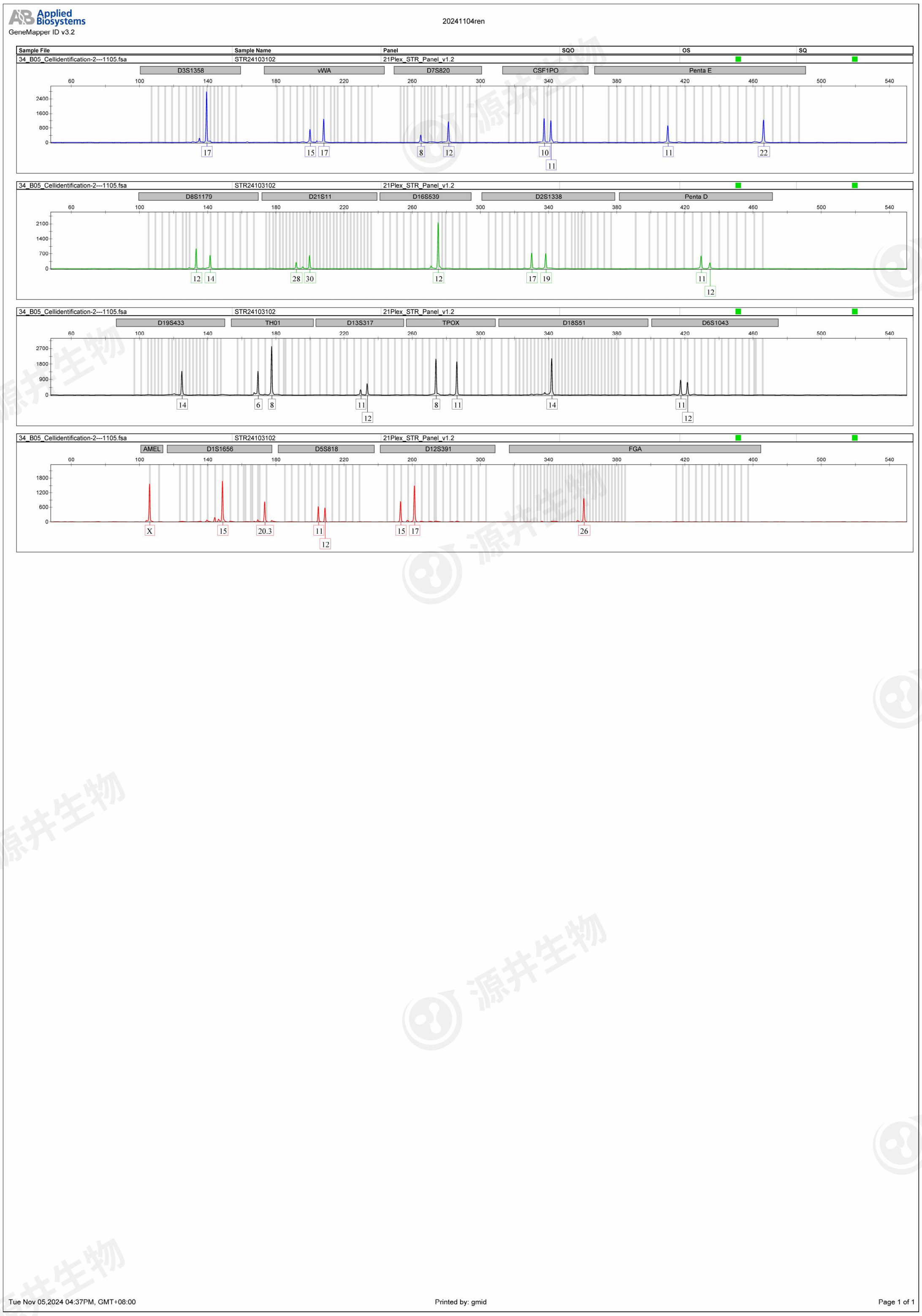
Applied Biosystems
GeneMapper ID v3.2
20241104ren
Sample File
Sample Name
Panel
SQO
OS
SQ
34_B05_Cellidentification-2---1105.fsa
STR24103102
21Plex_STR_Panel_v1.2
D3S1358
vWA
D7S820
CSF1PO
Penta E
17
15 17
8 12
10 11
11
22
D8S1179
D21S11
D16S539
D2S1338
Penta D
12 14
28 30
12
17 19
11 12
D19S433
TH01
D13S317
TPOX
D18S51
D6S1043
14
6 8
11 12
8 11
14
11 12
AMEL
D1S1656
D5S818
D12S391
FGA
X
15
20.3
11 12
15 17
26
Tue Nov 05,2024 04:37PM, GMT+08:00
Printed by: gmid
Page 1 of 1

细胞遗传质量鉴定检测

Cell Line Authentication Service

一、细胞名称：HK-2

二、检测方法：用 Axygen 的基因组抽提试剂盒提取 DNA，采用 21- STR 扩增方案扩增，在 ABI 3730XL 型遗传分析仪上对 STR 位点和性别基因 Amelogenin 进行检测。

三、样本基因分型结果：

细胞的 STR 位点和 Amelogenin 位点的基因分型结果						
Loci	送检细胞 STR 信息			细胞库细胞 STR 信息		
	送检细胞名：HK2-Y01-K			细胞库细胞名：HK-2 [Human kidney]		
	Allele1	Allele2	Allele3	Allele1	Allele2	Allele3
D5S818	12	12		12	12	
D13S317	9	9		9	9	
D7S820	10	11		10	11	
D16S539	11	12		11	12	
VWA	17	18		17	18	
TH01	9	9		9	9	
AMEL	X	X		X	X	
TPOX	8	9		8	9	
CSF1PO	13	13		13	13	
D12S391	18	22				
FGA	20	22				
D2S1338	17	25				
D21S11	28	30				
D18S51	12	12				
D8S1179	10	14				
D3S1358	16	17				
D6S1043	12	13				

PENTAE	10	11				
D19S433	15	15.2				
PENTAD	9	12				
D1S1656	12	13				

四、结论：**HK-2** 细胞 STR 分型结果与 **EXPASY** 数据库 **HK-2** 细胞系基因型一致，细胞号对应 **CVCL_0302**，STR 分型结果**完全匹配**。本次检测在该细胞系中**没有发现多等位基因**。

五、附图

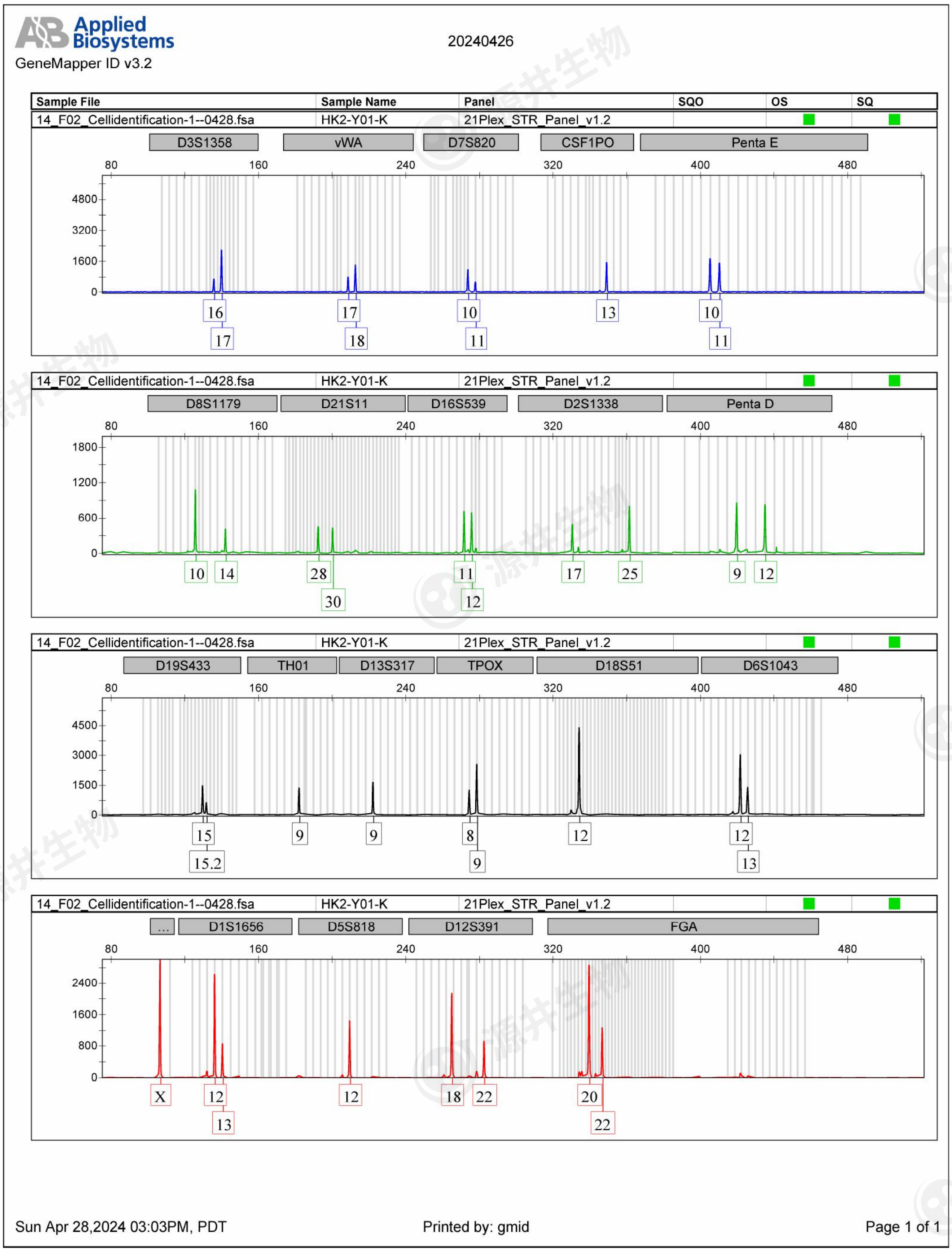
Applied Biosystems
20240426
GeneMapper ID v3.2
Sample File
Sample Name
Panel
SQO
OS
SQ
14_F02_Cellidentification-1--0428.fsa
HK2-Y01-K
21Plex_STR_Panel_v1.2
D3S1358
vWA
D7S820
CSF1PO
Penta E
16 17 17 18 10 11 13 10 11
D8S1179
D21S11
D16S539
D2S1338
Penta D
10 14 28 30 11 12 17 25 9 12
D19S433
TH01
D13S317
TPOX
D18S51
D6S1043
15 15.2 9 9 8 9 12 12 13
...
D1S1656
D5S818
D12S391
FGA
X 12 13 12 18 22 20 22
Sun Apr 28,2024 03:03PM, PDT
Printed by: gmid
Page 1 of 1

细胞遗传质量鉴定检验报告

检品名称：细胞系

送检单位：上海富衡生物科技有限公司

检验类型：STR 基因型检验

发出时间：2016 年 9 月 11 日

遗传质量鉴定检验报告

样品编号:

表 1 样本编号

客户样本编号	公司编号
031	20160902-02

样品数量： 1

样品性状： 细胞系

检测项目： STR

送检单位： **上海富衡生物科技有限公司**

检测方法： 用 Axygen 的基因组抽提试剂盒提取 DNA，采用 20- STR 扩增方案扩增，在 ABI 3730XL 型遗传分析仪上对 STR 位点和性别基因 Amelogenin 进行检测。

检验结果:

(一)检验基本情况

表 2：样本基因型检验结果

样本编号	多等位基因	匹配细胞系	细胞库	匹配说明
20160902-02	无	OS-RC-2	DSMZ	完全匹配

- 多等位基因指三等位及以上基因现象。
- 本次检测各细胞分型结果良好。

(二)各样本描述

- 20160902-02：该株细胞 DNA 分型在细胞系检索中找到完全匹配的细胞系，DSMZ 数据库显示细胞名为 OS-RC-2，细胞号对应 **RCB0735**。本次检测在该细胞系中**没有发现多等位基因**。

(三)样本分型结果

表 4：细胞 20160902-02 的 STR 位点和 Amelogenin 位点的基因分型结果

Marker	样本				细胞库信息		
	Allele1	Allele2	Allele3	Allele4	Allele1	Allele2	Allele3
D5S818	11	12			11	12	
D13S317	8	12			8	12	
D7S820	10	11			10	11	
D16S539	11	11			11	11	
VWA	16	16			16	16	
TH01	6	6			6	6	
AMEL	X	Y			X	Y	
TPOX	8	11			8	11	
CSF1PO	12	13			12	13	
D12S391	20	23					
FGA	24	24					
D2S1338	20	20					
D21S11	31.2	31.2					
D18S51	14	14					
D8S1179	14	14					
D3S1358	17	17					
D6S1043	12	12					
PENTAE	11	17					
D19S433	13	14					
PENTAD	11	11					

其他说明:

(一)分型方案及位点分布:

附表：实验方案及位点

	方案 1	方案 2	方案 3	方案 4	方案 5	方案 6
1	TH01	AMEL	TPOX	D3S1358	PENTAE	D19S433
2	D12S391	D5S818	VWA	D13S317		PENTAD
3	D7S820	D2S1338	D8S1179	D6S1043		
4	CSF1PO	D21S11		D16S539		
5	FGA	D18S51				

(二)STR 数据库比对:

本公司采用 DSMZ tools 进行细胞系比对，其中包含来自于 ATCC, DSMZ, JCRB 和 RIKEN 数据库的 2455 个细胞系 STR 数据。如果待检测细胞未收录于以上细胞库或这是自行建立的新细胞系将无法进行比对，用户需根据细胞分型结果自行与其他数据库进行比对。

签发日期：2016 年 9 月 11 日

Applied Biosystems
GeneMapper 4.0

Sample File	Sample Name	Panel	OS	SQ
13_E02_XiBaoJianDing(0905+0902)-1-0907.fsa	031	STR Profile 1-dup		

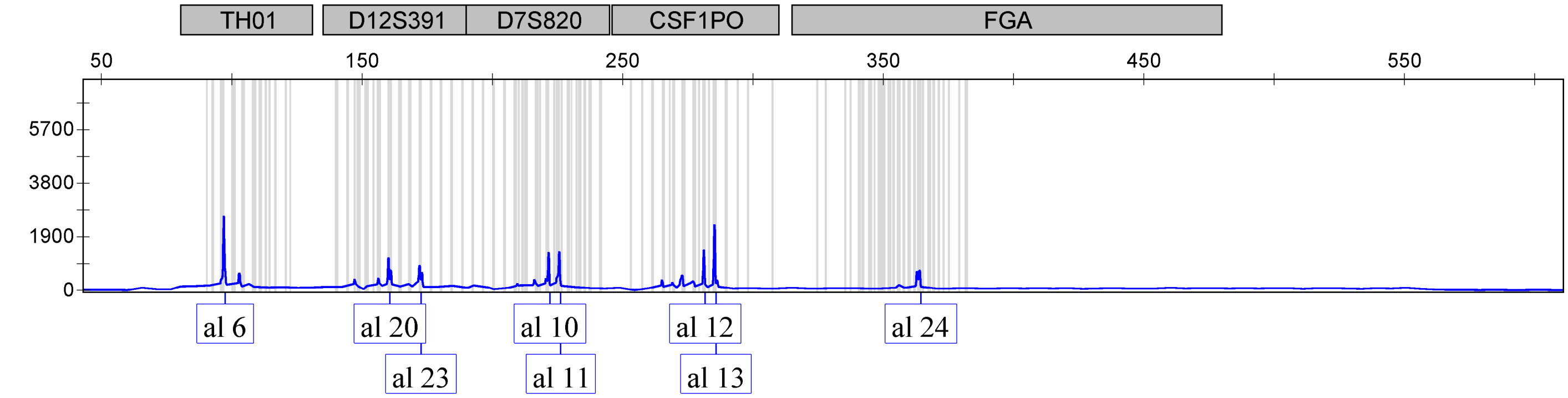

Sample File	Sample Name	Panel	OS	SQ
53_E07_XiBaoJianDing(0905+0902)-1-0907.fsa	031	STR Profile 2-dup		

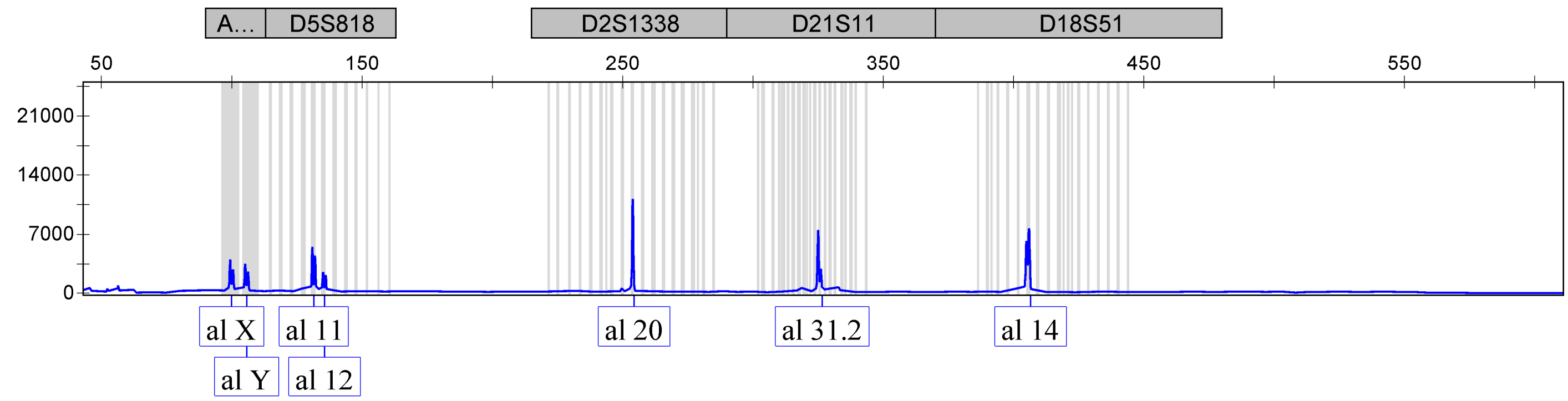

Sample File	Sample Name	Panel	OS	SQ
85_E11_XiBaoJianDing(0905+0902)-1-0907.fsa	031	STR Profile 3-dup		

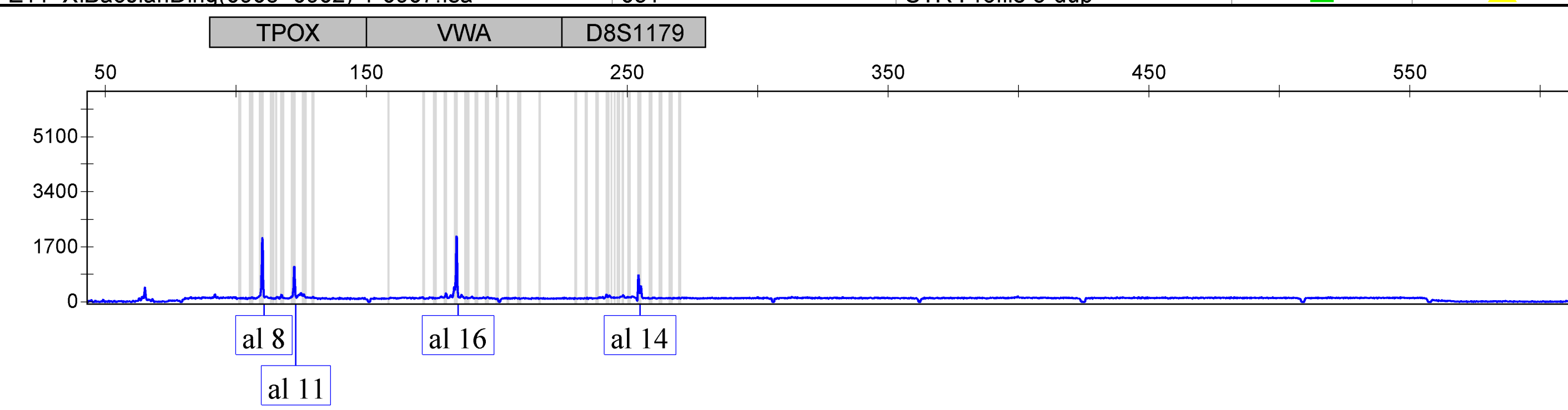

Applied Biosystems

GeneMapper 4.0

Cell Line Authentication-2

Sample File	Sample Name	Panel	OS	SQ
29 E04 XiBaoJianDing(0905+0902)-3-0907.FSA	031	STR Profile 4-dup		

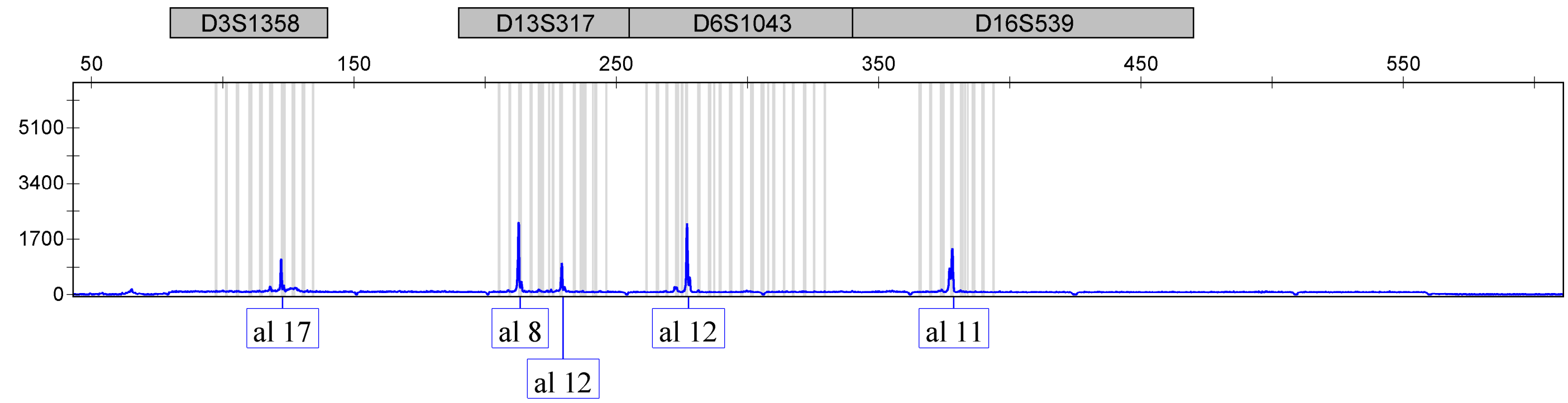

Sample File	Sample Name	Panel	OS	SQ
61 E08 XiBaoJianDing(0905+0902)-3-0907.FSA	031	STR Profile 5-dup		

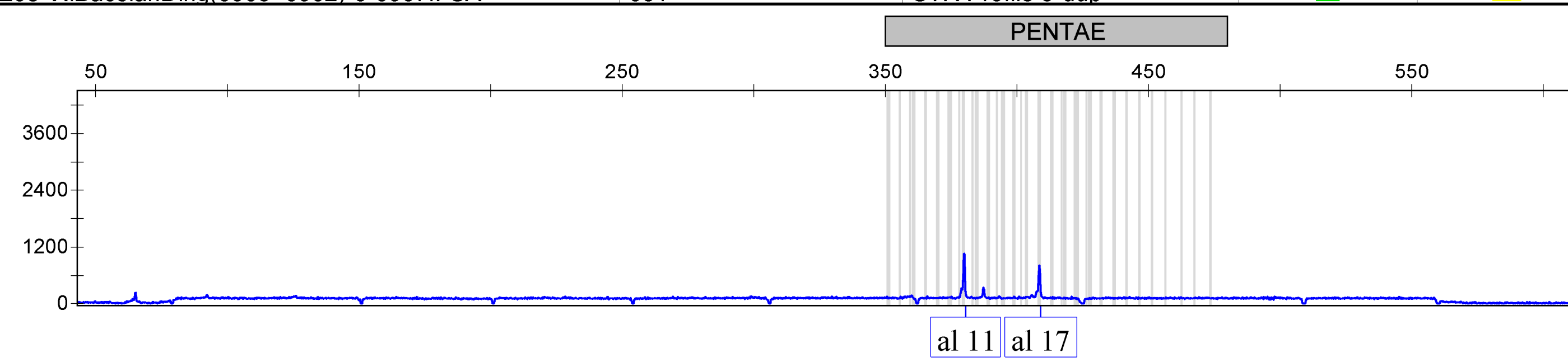

Sample File	Sample Name	Panel	OS	SQ
93 E12 XiBaoJianDing(0905+0902)-3-0907.FSA	031	STR Profile 6-dup		

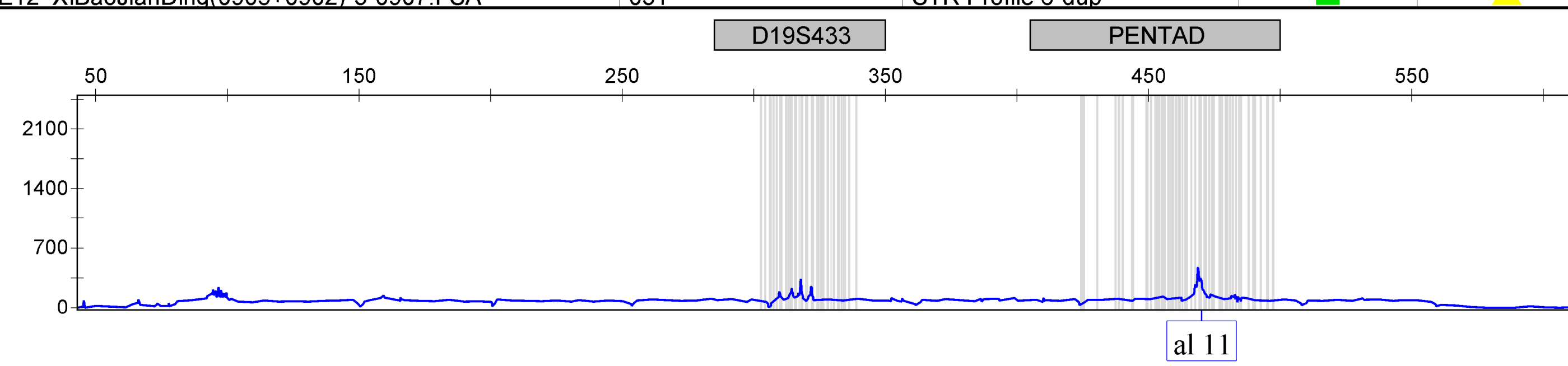

Sample File	Sample Name	Panel	OS	SQ
32_H04_Xia(1-2)1-4(3-2)5-8-0909.fsa	031	STR Profile 6-dup		

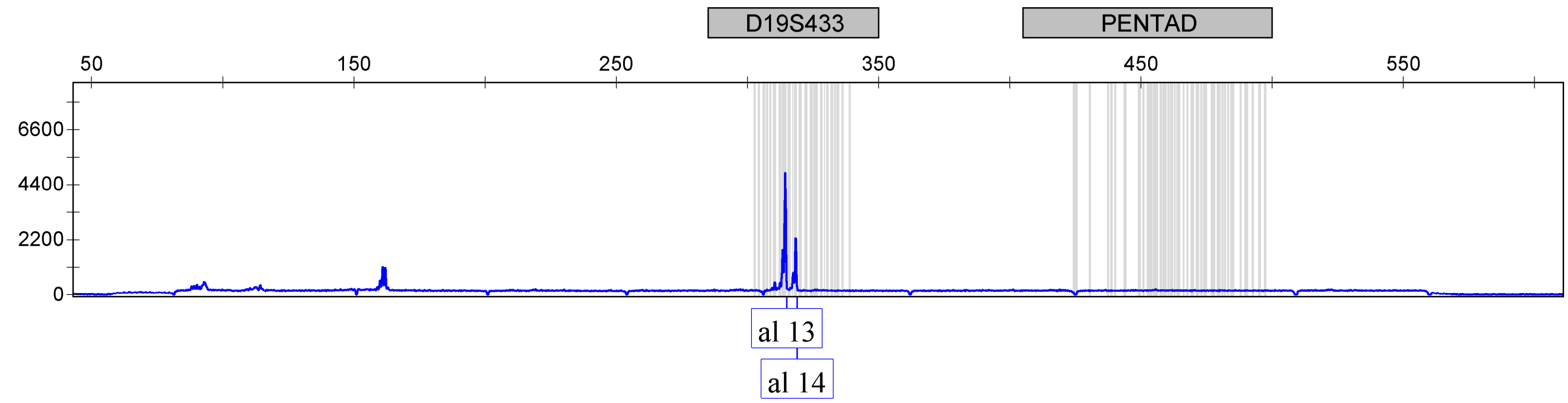

Supplement: Supplementary file 1 [file CTM2-16-e70880-s011.pdf]
